# Supplementary material for: A novel method for multiple phenotype association studies based on genotype and phenotype network
Source: PLoS Genet. 2024 May 10;20(5):e1011245. doi: 10.1371/journal.pgen.1011245 (PMC11111089; doi:10.1371/journal.pgen.1011245)
Supplement: S1 Text — Text A. Details of the six multiple phenotype association tests. Text B. Details of simulation models. Text C. Comparison of methods in the simulation studies. Text D. The preprocess of genotype and phenotype in UK Biobank. Table A-F. The estimated type I error rates of the six multiple phenotype association tests divided by the nominal significance level for 60, 80, and 100 mixture phenotypes (half continuous phenotypes and half binary phenotypes with balanced case-control ratios) under models 1–6. The type I error rates are evaluated using 500 MC runs (equivalent to 106 replicates). Table G-L. The estimated type I error rates of the six multiple phenotype association tests divided by the nominal significance level for 60, 80, and 100 binary phenotypes (with extremely unbalanced case-control ratios) under models 1–6. The type I error rates are evaluated using 500 MC runs (equivalent to 106 replicates). Table M. The estimated type I error rates of the six multiple phenotype association tests divided by the nominal significance level for 60, 80, and 100 mixture phenotypes (half continuous phenotypes and half binary phenotypes with balanced case-control ratios) under null simulation (no clusters/categories of phenotypes). The type I error rates are evaluated using 500 MC runs (equivalent to 106 replicates). Table N. 33 unique SNPs identified by ceCLC for testing the association in NET (one SNP) or in N.O. (32 SNPs). Table O. ICD-10 codes and names of the14 reported diseases shown in Table N. Table P. Simulation settings for the six models with λ^1=β(1,⋯,1)T and λ^2=2βk+1(1,⋯,k)T. Fig A. Power comparisons of the six tests as a function of effect size β under six models. The number of mixture phenotypes (half continuous phenotypes and half binary phenotypes with balanced case-control ratios) is 80 and the sample size is 2,000. The power of all of the six tests is evaluated using 10 MC runs. Fig B. Power comparisons of the six tests as a function of effect size β under s [file pgen.1011245.s001.docx]

**Supplementary Materials**

**A novel method for multiple phenotype association studies based on genotype and phenotype network**

Xuewei Cao^1^, Shuanglin Zhang^1^, Qiuying Sha^1,*^

^1^Department of Mathematical Sciences, Michigan Technological University, Houghton, Michigan, USA

Xuewei Cao, Department of Mathematical Sciences, Michigan Technological University, Houghton, Michigan 49931, USA. E-mail: [xueweic@mtu.edu](mailto:xueweic@mtu.edu)

Shuanglin Zhang, Department of Mathematical Sciences, Michigan Technological University, Houghton, Michigan 49931, USA. E-mail: [shuzhang@mtu.edu](mailto:shuzhang@mtu.edu)

**^*^Corresponding author**: Qiuying Sha, Department of Mathematical Sciences, Michigan Technological University, Houghton, Michigan 49931, USA. E-mail: [qsha@mtu.edu](mailto:qsha@mtu.edu)

**Text A. Details of the six multiple phenotype association tests.**

To test the association between phenotypes in each network module and a SNP, we perform the following six multiple phenotypes association tests. To simplify the notation, we assume that these tests are applied to test the association between $K$ phenotypes and a SNP.

***CLC*** [1]: CLC classifies $K$ phenotypes into $L$ clusters for $L=1,\cdots,K$. The test statistic with $L$ clusters is given by $T_{CLC}^{L}=\left(\mathbf{D}T\right)^{T}\left(\mathbf{D\Sigma D}^{T}\right)^{-1}\left(\mathbf{D}T\right)$, where $T=\left(T_{1},\cdots,T_{K}\right)^{T}$ with $T_{k}$ being the score test statistic to test the association between the $k^{th}$ phenotype and a SNP; $\mathbf{D}=\mathbf{B}^{T}\mathbf{\Sigma}^{-1}$ with $\mathbf{\Sigma}$ being a correlation matrix of $K$ phenotypes and $\mathbf{B}=\left(b_{kl}\right)$ being a $K\times L$ matrix with $b_{kl}=1$ if the $k^{th}$ phenotype belongs to the $l^{th}$ cluster and $b_{kl}=0$ otherwise. Under the null hypothesis that none of the $K$ phenotypes are associated with a SNP, $T_{CLC}^{L}$ follows a chi-square distribution with degrees of freedom $L$. The overall test statistic of CLC is given by $T_{CLC}=\max_{1\leq L\leq K} T_{CLC}^{L}$ and the corresponding p-value can be evaluated by a simulation procedure.

***ceCLC*** [2]: ceCLC is a computational efficient version of CLC, where the p-value of overall test statistic is derived by the Cauchy combination method [3, 4]. Let $p_{L}$ be the p-value of $T_{CLC}^{L}$, then the test statistic of ceCLC is given by $T_{ceCLC}=\sum_{L=1}^{K} \tan\left\{\left(0.5-p_{L}\right)\pi\right\}/K$. The null distribution of $T_{ceCLC}$ can be well approximated by a standard Cauchy distribution. Therefore, the p-value of ceCLC can be approximated by $p_{ceCLC}=0.5-\left\{\arctan\left(T_{ceCLC}\right)/\pi\right\}$.

***HCLC*** [5]: Instead of considering all possible number of clusters in CLC and ceCLC, HCLC determine the optimal number of clusters, $L^{*}$, by using a stopping criterion that maximizes the cluster separation [6]. Therefore, the test statistic of HCLC is defined as $T_{HCLC}=T_{CLC}^{L^{*}}$ and the p-value is calculated by assuming $T_{HCLC}$ follows a chi-square distribution with degrees of freedom $L^{*}$.

***MultiPhen*** [7]: MultiPhen uses the ordinal regression (also known as proportional odds logistic regression) to regress genotype of a SNP on $K$ phenotypes. MultiPhen uses a likelihood ratio test to test whether effect sizes of $K$ phenotypes are significantly different from zero. The resulting test statistic asymptotically follows a chi-square distribution with degrees of freedom $K$.

***O’Brien*** [8]: O’Brien uses a linear combination method of the score test statistic, $T_{k}$ , to test the association between the $k^{th}$ phenotype and a SNP. That is, the test statistic of O’Brien is given by $T_{O'Brien}=\left(1_{K}^{T}\mathbf{\Sigma}^{-1}T\right)^{2}$, where $1_{K}$ is a $K\times1$ vector with elements of all 1s. Under the null hypothesis, $T_{O'Brien}$ follows a chi-square distribution with 1 degree of freedom.

**Omnibus** [1]: Omnibus is developed to overcome the limitation of O’Brien. The test statistic of Omnibus is $T_{omnibus}=T^{T}\mathbf{\Sigma}^{-1}T$. Under the null hypothesis, $T_{omnibus}$ follows a chi-square distribution with degrees of freedom $K$.

Note that a normal approximation of the score test statistic $T_{k}$ for $k=1,\cdots,K$ used in CLC, ceCLC, HCLC, O’Brien, and Omnibus has inflated type I error rates from binary phenotypes with extremely unbalanced case-control ratios [9]. In this case, we modify these five methods by calculating $T_{k}=\hat{\beta}_{k}/\widehat{se}\left(\hat{\beta}_{k}\right)$, where $\hat{\beta}_{k}$ and $\widehat{se}\left(\hat{\beta}_{k}\right)$ can be estimated by saddplepoint approximation [9].

**Text B. Details of simulation models.**

We consider the following six simulation models (**Table O**) with $M=2,000$, $MAF\sim U\left(0.05,0.5\right)$, $\rho=0.3$, $c_{0}^{2}=0.5$, and $\rho_{f}=0.3/c_{0}^{2}$ (between-category correlation is 0.3). $\hat{\lambda}_{1}=\beta\left(1,\cdots,1\right)^{T}$ and $\hat{\lambda}_{2}=\frac{2\beta}{k+1}\left(1,\cdots,k\right)^{T}$ are two types of effect sizes.

Model 1: $M_{\text{causal}}=100$, $C=2$, and all phenotypes are associated with at least one SNP with the same effect sizes but different directions. That is, the first 50 SNPs affect the phenotypes in the first category with $\hat{\lambda}_{1}$ and the second 50 SNPs affect the phenotypes in the second category with $-\hat{\lambda}_{1}$.

Model 2: $M_{\text{causal}}=100$, $C=2$, and all phenotypes are associated with at least one SNP with different effect sizes and different directions. That is, the first 50 SNPs affect the phenotypes in the first category with $\hat{\lambda}_{1}$ and the second 50 SNPs impact the phenotypes in the second category with $-\hat{\lambda}_{2}$.

Model 3: $M_{\text{causal}}=100$, $C=5$, and only phenotypes in the first two categories are associated with the first 100 SNPs with the same settings as in Model 1. The phenotypes in the remaining three categories do not associate with any SNPs.

Model 4: $M_{\text{causal}}=100$, $C=5$, and only phenotypes in the first two categories are associated with the first 100 SNPs with the same settings as in Model 2. The phenotypes in the remaining three categories do not associate with any SNPs.

Model 5: $M_{\text{causal}}=200$, $C=4$, and all phenotypes are associated with at least one SNP. That is, the first 50 SNPs affect the phenotypes in the first category with $\hat{\lambda}_{1}$, the second 50 SNPs affect the phenotypes in the second category with $-\hat{\lambda}_{1}$, the third 50 SNPs affect the phenotypes in the third category with $\hat{\lambda}_{2}$, and the fourth 50 SNPs affect the phenotypes in the fourth category with $-\hat{\lambda}_{2}$.

Model 6: $M_{\text{causal}}=200$, $C=10$, and only phenotypes in the first four categories are associated with the first 200 SNPs with the same settings as in Model 5. The phenotypes in the remaining six categories do not associate with any SNPs.

In our simulation studies, we also consider a more realistic null simulation setup (No cluster model). Consider there is no clusters of $K$ phenotypes. That is, $C=K, \text{ and }\rho=0$. To mimic the cluster structure from a real dataset, we also consider a simulaiton model based on the information obtained from the UK Biobank. We consider $K=52$ phenotypes in the UK Biobank with both genetic correaltion and phenotypic correlation estimated by the LD score regression (LDSC) (*More details in Correlation Analysis section*). Let $\mathbf{\Sigma}_{g}$ and $\mathbf{\Sigma}_{p}$ be the genetic correlation and phenotypic correlation of $K=52$ phenotypes estimated by LDSC. Consider $M_{\text{causal}}=100$ causal variants out of $M=2,000$ variants in each MC run. We generate $\mathbf{B}_{causal}\sim MVN_{K}\left(0,h^{2}\mathbf{\Sigma}_{g}\right)$ with $h^{2}=0.05$. Therefore, the quantitative phenotypes can be generated by $\mathbf{Y}=\mathbf{G}_{causal}\mathbf{B}_{causal}+\mathbf{E}$, where $\mathbf{E}\sim MVN_{K}\left(0,\mathbf{\Sigma}_{p}\right)$ and $\mathbf{G}_{causal}=\left(G_{1},\cdots,G_{M_{causal}}\right)$ is the matrix of $M_{causal}$ SNPs with dimension $N\times M_{causal}$. Each $G_{i}$ is generated from a distribution. Then, we random change half of quantitative phenotypes to qualitative phenotypes by using the liability threshold model. Here, we consider $N=2,000$ and $4,000$in our simulation model.

**Text C. Comparison of methods in the simulation studies.**

We use six multiple phenotype association tests to evaluate the performance of our proposed method based on network modules. Therefore, we consider the following two types of comparisons.

*Comparison 1: Apply* *six multiple phenotype association tests without considering network modules.*

We test the association between $K$ phenotypes and a SNP. For each simulation model, we run $B$ Monte-Carlo (MC) runs. The steps for the $b^{th}$ MC run are as follow. i). Generate $N$ individuals with $M$ SNPs and $K$ phenotypes in $C$ categories; ii). Test the association between $K$ phenotypes and $M$ SNPs using each of the multiple phenotype association tests. The p-value for the $m^{th}$ SNP in the $b^{th}$ MC run is given by $p_{m}^{\left(b\right)}$. To evaluate the type I error rates of the tests, we generate phenotypes from the null model, that is, for each model, we set $\beta=0$. The type I error rate, $\text{T1E}_{N.O.}$, can be calculated by

$$\text{T1E}_{N.O.}=\frac{\sum_{b=1}^{B} \sum_{m=1}^{M} I\left(p_{m}^{\left(b\right)}\leq\alpha\right)}{B\times M}.$$

To evaluate power, we generate phenotypes from each of the six models with different effect sizes $\beta$. The power, $\text{power}_{N.O.}$, can be calculated by

$$\text{power}_{N.O.}=\frac{\sum_{b=1}^{B} \sum_{m=1}^{M_{\text{causal}}} I\left(p_{m}^{\left(b\right)}\leq\alpha\right)}{B\times M_{\text{causal}}}.$$

*Comparison 2:* *Apply six multiple phenotype association tests by considering network modules.*

For each simulation model, we run $B$ MC runs. We use the following steps for $b^{th}$ MC run. i). Generate $N$ individuals with $M$ SNPs and $K$ phenotypes in $C$ categories; ii). Construct the GPN based on the shared genetic architecture; iii). Detect $C^{\left(b\right)}$ network modules for the $K$ phenotypes using the community detection method; iv). Test the association between phenotypes in each of the $C^{\left(b\right)}$ network modules and each of $M$ SNPs using one of the six tests. We use $p_{cm}^{\left(b\right)}$ to denote the p-value of the assocition test between phenotypes in the $c^{th}$ network module and the $m^{th}$ SNP for $c=1,2,\cdots,C^{(b)}$. To evaluate the type I error rate of a test based on the network modules, we generate phenotypes under the null model. That is, for each model, we set $\beta=0$. The type I error rate, $\text{T1E}_{NET}$, can be calculated by

$$\text{T1E}_{NET}=\frac{\sum_{b=1}^{B} \sum_{m=1}^{M} I\left(\min_{c=1,\cdots,C^{\left(b\right)}} \left\{p_{cm}^{\left(b\right)}\right\}\leq\alpha/C^{\left(b\right)}\right)}{B\times M}.$$

To evaluate power, we generate phenotypes for each model with different effect sizes $\beta$. The power, $\text{power}_{NET}$, can be calculated by

$$\text{power}_{NET}=\frac{\sum_{b=1}^{B} \sum_{m=1}^{M_{\text{causal}}} I\left(\min_{c=1,\cdots,C^{\left(b\right)}} \left\{p_{cm}^{\left(b\right)}\right\}\leq\alpha/C^{\left(b\right)}\right)}{B\times M_{\text{causal}}}.$$

**Text D. The preprocess of genotype and phenotype in UK Biobank**

Genotypes from the UK Biobank have extracted 488,377 participants with 784,256 variants in autosomal chromosomes. The preprocess of genotype is achieved by quality controls (QCs) which are performed on both SNPs and individuals using PLINK 1.9 [10]. Same QC steps as Liang et al. [5] (**Figure R**), we filter out SNPs with missing rates larger than 5%, Hardy-Weinberg equilibrium exact test p-values less than $10^{-6}$, and MAF less than 5%. We also filter out individuals with missing rates larger than 5% and individuals without sex. After quality controls, 288,647 SNPs and 466,580 individuals remain for our next step analysis.

In this study, we define EHR-derived phenotypes using the ICD-10 codes, which is a standardized coding system for defining disease status as well as for billing purposes [11]. After truncating each full ICD-10 code to UK Biobank ICD-10 level 3 code, we consider 72 unique truncated ICD codes with the number of cases greater than 200 in Chapter XIII (Diseases of the musculoskeletal system and connective tissue), such as rheumatoid arthritis (M06.9), psoriatic arthropathies (M07.3), and gout (M10.9), etc. Note that there are two phenotypes (M45: Ankylosing spondylitis and M45.X9: Ankylosing spondylitis (Site unspecified)) which are not truncated by the ICD-10 code digits, however, these two phenotypes are defined by UK Biobank level 3 code. For each individual, if a corresponding truncated ICD code ever appears, we denote the EHR-derived phenotype for that individual as “1”, otherwise, we denote the EHR-derived phenotype for that individual as “0”. After truncating ICD-10 codes, we generate a total of 502,591 individuals who have 72 EHR-derived phenotypes in Chapter XIII. Following the phenotype preprocess introduced in Liang et al. [5], 337,285 individuals are kept (**Figure R**).

After data preprocessing procedures, individuals with both genotype and phenotype information are used in our study. There is a complete set of 322,607 individuals across 288,647 SNPs with 72 EHR-derived phenotypes. Among the 72 phenotypes, lumbar and other intervertebral disk disorders with myelopathy (M51.0) has the smallest case-control ratio 0.000658 with 212 cases and 322,395 controls; Gonarthrosis (M17.9) has the largest case-control ratio 0.03937 with 12,218 cases and 310,389 controls. Therefore, all of the phenotypes we considered in our analysis have extremely unbalanced case-control ratios. Furthermore, each phenotype is adjusted by 13 covariates, including age, sex, genotyping array, and the first 10 genetic principal components (PCs) [12]. The analysis is performed based on the adjusted phenotypes.

**Table A**. The estimated type I error rates of the six multiple phenotype association tests divided by the nominal significance level for 60, 80, and 100 mixture phenotypes (half continuous phenotypes and half binary phenotypes with balanced case-control ratios) under model 1. The type I error rates are evaluated using 500 MC runs (equivalent to $10^{6}$ replicates).

| **Mixture Phenotypes**  **Model 1** | | | **ceCLC** | | **CLC** | | **HCLC** | | **MultiPhen** | | **O’Brien** | | **Omnibus** | |
| --- | --- | --- | --- | --- | --- | --- | --- | --- | --- | --- | --- | --- | --- | --- |
| $K$ | Sample | $\alpha$-level | N.O. | NET | N.O. | NET | N.O. | NET | N.O. | NET | N.O. | NET | N.O. | NET |
| 60 | 2000 | 0.001 | 0.959 | 0.992 | 0.889 | 0.978 | 0.798 | 0.964 | **1.408** | **1.120** | 0.995 | 0.991 | 0.796 | 0.863 |
|  |  | 0.0001 | 0.830 | 0.920 | 0.760 | 0.940 | 0.730 | 0.910 | **1.500** | **1.240** | 1.040 | 1.050 | 0.650 | 0.880 |
|  | 4000 | 0.001 | **1.087** | **1.094** | 1.014 | 1.005 | 0.944 | 1.001 | **1.282** | **1.142** | 1.061 | 1.001 | 0.958 | 1.051 |
|  |  | 0.0001 | 1.080 | 1.040 | 0.950 | 1.080 | 1.020 | 1.140 | **1.450** | 1.130 | 1.050 | 0.990 | 1.020 | 1.070 |
| 80 | 2000 | 0.001 | 0.926 | 0.970 | 0.839 | 0.931 | 0.801 | 0.957 | **1.790** | **1.279** | 0.995 | 0.972 | 0.775 | 0.842 |
|  |  | 0.0001 | 0.760 | 0.850 | 0.900 | 1.020 | 0.680 | 0.990 | **1.960** | **1.260** | 0.930 | 0.930 | 0.640 | 0.750 |
|  | 4000 | 0.001 | 1.061 | 1.031 | 0.960 | 0.952 | 0.884 | 0.975 | **1.388** | **1.166** | 0.985 | 0.936 | 0.894 | 0.980 |
|  |  | 0.0001 | 1.090 | 1.040 | 0.930 | 1.120 | 0.790 | 1.090 | **1.350** | 1.160 | 1.040 | 0.910 | 0.810 | 1.020 |
| 100 | 2000 | 0.001 | 0.902 | 0.943 | 0.800 | 0.889 | 0.703 | 0.930 | **2.147** | **1.291** | 0.935 | 0.927 | 0.706 | 0.815 |
|  |  | 0.0001 | 0.870 | 0.790 | 0.840 | 0.850 | 0.610 | 0.830 | **2.440** | **1.500** | 0.790 | 0.860 | 0.620 | 0.870 |
|  | 4000 | 0.001 | 0.985 | 1.032 | 0.940 | 0.970 | 0.887 | 0.977 | **1.523** | **1.155** | 0.941 | 1.003 | 0.890 | 0.946 |
|  |  | 0.0001 | 0.890 | 0.980 | 1.010 | 1.100 | 0.630 | 1.030 | **1.390** | 1.110 | 1.110 | 1.010 | 0.710 | 0.880 |

*Notes:* bold-faced values indicate that the values are beyond the upper bounds of the 95% CIs. 95% CIs for type I error rates divided by nominal significance levels 0.001 and 0.0001 are (0.938, 1.062) and (0.804, 1.196), respectively. “N.O.” represents the type I error rates calculated by the formula in Comparison 1 (Apply methods without considering network modules.). “NET” presents the type I error rates evaluated by the formula in Comparison 2 (Apply methods by considering network modules).

**Table B**. The estimated type I error rates of the six multiple phenotype association tests divided by the nominal significance level for 60, 80, and 100 mixture phenotypes (half continuous phenotypes and half binary phenotypes with balanced case-control ratios) under model 2. The type I error rates are evaluated using 500 MC runs (equivalent to $10^{6}$ replicates).

| **Mixture Phenotypes**  **Model 2** | | | **ceCLC** | | **CLC** | | **HCLC** | | **MultiPhen** | | **O’Brien** | | **Omnibus** | |
| --- | --- | --- | --- | --- | --- | --- | --- | --- | --- | --- | --- | --- | --- | --- |
| $K$ | Sample | $\alpha$-level | N.O. | NET | N.O. | NET | N.O. | NET | N.O. | NET | N.O. | NET | N.O. | NET |
| 60 | 2000 | 0.001 | 0.968 | 0.988 | 0.899 | 0.936 | 0.851 | 0.956 | **1.501** | **1.206** | 1.006 | 0.974 | 0.848 | 0.909 |
|  |  | 0.0001 | 0.910 | 0.840 | 0.870 | 0.870 | 0.780 | 1.060 | **1.540** | **1.300** | 1.080 | 0.940 | 0.790 | 0.810 |
|  | 4000 | 0.001 | 1.047 | 1.057 | 0.976 | 0.969 | 0.930 | 0.993 | **1.230** | **1.122** | 0.984 | 0.972 | 0.915 | 0.977 |
|  |  | 0.0001 | 0.960 | 1.010 | 1.030 | 1.000 | 1.000 | 1.000 | **1.380** | 1.180 | 1.110 | 1.000 | 0.970 | 1.080 |
| 80 | 2000 | 0.001 | 0.894 | 0.997 | 0.841 | 0.967 | 0.757 | 0.944 | **1.738** | **1.253** | 1.051 | 1.018 | 0.751 | 0.847 |
|  |  | 0.0001 | 0.810 | 1.010 | 0.730 | 0.880 | 0.760 | 0.890 | **1.990** | **1.580** | 1.160 | 1.150 | 0.770 | 0.950 |
|  | 4000 | 0.001 | 0.967 | 0.992 | 0.965 | 0.912 | 0.879 | 0.955 | **1.300** | **1.095** | 1.030 | 1.011 | 0.861 | 0.908 |
|  |  | 0.0001 | 0.980 | 0.970 | 0.840 | 0.920 | 0.700 | 0.980 | **1.270** | 1.120 | 0.990 | 0.970 | 0.780 | 0.740 |
| 100 | 2000 | 0.001 | 0.858 | 0.939 | 0.821 | 0.885 | 0.707 | 0.908 | **2.128** | **1.344** | 1.015 | 0.930 | 0.704 | 0.782 |
|  |  | 0.0001 | 0.810 | 0.960 | 0.730 | 0.880 | 0.570 | 1.090 | **2.390** | **1.430** | 0.980 | 0.850 | 0.610 | 0.790 |
|  | 4000 | 0.001 | 0.995 | 0.984 | 0.886 | 0.962 | 0.881 | 0.980 | **1.461** | **1.193** | 1.046 | 0.957 | 0.870 | 0.922 |
|  |  | 0.0001 | 1.080 | 0.990 | 0.970 | 0.890 | 0.930 | 0.990 | **1.900** | 1.070 | 1.030 | 1.040 | 0.910 | 0.760 |

*Notes:* bold-faced values indicate that the values are beyond the upper bounds of the 95% CIs. 95% CIs for type I error rates divided by nominal significance levels 0.001 and 0.0001 are (0.938, 1.062) and (0.804, 1.196), respectively. “N.O” represents the type I error rates calculated by the formula in Comparison 1 (Apply methods without considering network modules.). “NET” presents the type I error rates evaluated by the formula in Comparison 2 (Apply methods by considering network modules).

**Table C**. The estimated type I error rates of the six multiple phenotype association tests divided by the nominal significance level for 60, 80, and 100 mixture phenotypes (half continuous phenotypes and half binary phenotypes with balanced case-control ratios) under model 3. The type I error rates are evaluated using 500 MC runs (equivalent to $10^{6}$ replicates).

| **Mixture Phenotypes**  **Model 3** | | | **ceCLC** | | **CLC** | | **HCLC** | | **MultiPhen** | | **O’Brien** | | **Omnibus** | |
| --- | --- | --- | --- | --- | --- | --- | --- | --- | --- | --- | --- | --- | --- | --- |
| $K$ | Sample | $\alpha$-level | N.O. | NET | N.O. | NET | N.O. | NET | N.O. | NET | N.O. | NET | N.O. | NET |
| 60 | 2000 | 0.001 | 0.958 | 1.004 | 0.893 | 0.997 | 0.826 | 0.919 | **1.481** | **1.155** | 1.014 | 1.073 | 0.803 | 0.941 |
|  |  | 0.0001 | 0.880 | 1.000 | 0.800 | 1.170 | 0.620 | 0.990 | **1.530** | **1.230** | 0.930 | 1.170 | 0.610 | 0.790 |
|  | 4000 | 0.001 | 1.030 | 1.052 | 0.941 | 0.940 | 0.909 | 0.976 | **1.210** | **1.114** | 0.984 | 1.064 | 0.903 | 0.981 |
|  |  | 0.0001 | 0.700 | 1.050 | 0.840 | 1.010 | 0.840 | 1.080 | **1.420** | 1.180 | 1.010 | 1.210 | 0.960 | 0.840 |
| 80 | 2000 | 0.001 | 0.949 | 0.952 | 0.843 | 0.909 | 0.769 | 0.882 | **1.741** | **1.194** | 1.009 | **1.099** | 0.739 | 0.903 |
|  |  | 0.0001 | 0.710 | 0.850 | 0.790 | 0.760 | 0.580 | 0.740 | **2.060** | **1.250** | 0.990 | 1.070 | 0.580 | 0.740 |
|  | 4000 | 0.001 | 1.006 | 0.965 | 0.903 | 0.912 | 0.881 | 0.907 | **1.358** | **1.131** | 0.915 | 1.000 | 0.887 | 0.951 |
|  |  | 0.0001 | 0.890 | 0.920 | 0.900 | 1.070 | 0.810 | 0.910 | **1.470** | 1.070 | 0.840 | 1.090 | 0.850 | 1.020 |
| 100 | 2000 | 0.001 | 0.907 | 0.946 | 0.835 | 0.911 | 0.767 | 0.884 | **2.171** | **1.383** | 0.979 | **1.071** | 0.751 | 0.892 |
|  |  | 0.0001 | 0.830 | 0.890 | 0.670 | 0.810 | 0.740 | 0.780 | **2.760** | **1.430** | 0.840 | 1.070 | 0.680 | 0.770 |
|  | 4000 | 0.001 | 1.005 | 0.961 | 0.902 | 0.967 | 0.833 | 0.925 | **1.393** | **1.170** | 0.977 | 1.038 | 0.797 | 0.927 |
|  |  | 0.0001 | 0.900 | 0.730 | 0.950 | 0.950 | 0.630 | 0.770 | **1.410** | 0.940 | 0.960 | 1.060 | 0.650 | 0.710 |

*Notes:* bold-faced values indicate that the values are beyond the upper bounds of the 95% CIs. 95% CIs for type I error rates divided by nominal significance levels 0.001 and 0.0001 are (0.938, 1.062) and (0.804, 1.196), respectively. “N.O” represents the type I error rates calculated by the formula in Comparison 1 (Apply methods without considering network modules.). “NET” presents the type I error rates evaluated by the formula in Comparison 2 (Apply methods by considering network modules).

**Table D**. The estimated type I error rates of the six multiple phenotype association tests divided by the nominal significance level for 60, 80, and 100 mixture phenotypes (half continuous phenotypes and half binary phenotypes with balanced case-control ratios) under model 4. The type I error rates are evaluated using 500 MC runs (equivalent to $10^{6}$ replicates).

| **Mixture Phenotypes**  **Model 4** | | | **ceCLC** | | **CLC** | | **HCLC** | | **MultiPhen** | | **O’Brien** | | **Omnibus** | |
| --- | --- | --- | --- | --- | --- | --- | --- | --- | --- | --- | --- | --- | --- | --- |
| $K$ | Sample | $\alpha$-level | N.O. | NET | N.O. | NET | N.O. | NET | N.O. | NET | N.O. | NET | N.O. | NET |
| 60 | 2000 | 0.001 | 0.935 | 0.983 | 0.872 | 0.982 | 0.783 | 0.923 | **1.417** | **1.098** | 0.988 | **1.071** | 0.789 | 0.907 |
|  |  | 0.0001 | 0.890 | 0.980 | 0.930 | 0.800 | 0.650 | 0.950 | **1.460** | **1.260** | 0.980 | **1.370** | 0.690 | 0.850 |
|  | 4000 | 0.001 | 1.019 | 0.979 | 0.915 | 0.959 | 0.923 | 0.943 | **1.183** | **1.084** | 0.975 | 1.060 | 0.870 | 0.935 |
|  |  | 0.0001 | 1.120 | 1.090 | 0.870 | 0.890 | 0.940 | 1.020 | **1.320** | 1.190 | 0.940 | 0.990 | 0.860 | 1.000 |
| 80 | 2000 | 0.001 | 0.905 | 0.974 | 0.910 | 0.923 | 0.785 | 0.891 | **1.778** | **1.250** | 1.028 | 0.994 | 0.789 | 0.900 |
|  |  | 0.0001 | 0.860 | 0.860 | 0.760 | 0.880 | 0.550 | 0.910 | **1.870** | **1.290** | 1.020 | 1.170 | 0.510 | 0.820 |
|  | 4000 | 0.001 | 1.018 | 1.024 | 0.936 | 0.983 | 0.887 | 1.005 | **1.338** | **1.116** | 1.012 | 1.024 | 0.887 | 0.957 |
|  |  | 0.0001 | 0.870 | 0.940 | 0.790 | 0.980 | 1.000 | 0.990 | **1.700** | 1.060 | 0.970 | 1.090 | 1.050 | 0.860 |
| 100 | 2000 | 0.001 | 0.902 | 0.996 | 0.851 | 0.909 | 0.739 | 0.918 | **2.186** | **1.311** | 0.981 | 1.022 | 0.750 | 0.858 |
|  |  | 0.0001 | 1.000 | 0.890 | 0.830 | 1.010 | 0.680 | 0.810 | **2.750** | **1.450** | 0.840 | 1.020 | 0.770 | 0.680 |
|  | 4000 | 0.001 | 1.000 | 1.043 | 0.902 | 0.957 | 0.855 | 0.969 | **1.537** | **1.148** | 0.989 | 1.027 | 0.876 | 0.933 |
|  |  | 0.0001 | 0.980 | 1.040 | 1.000 | 1.030 | 0.970 | 1.070 | **1.760** | 1.180 | 0.980 | 1.020 | 1.000 | 1.030 |

*Notes:* bold-faced values indicate that the values are beyond the upper bounds of the 95% CIs. 95% CIs for type I error rates divided by nominal significance levels 0.001 and 0.0001 are (0.938, 1.062) and (0.804, 1.196), respectively. “N.O” represents the type I error rates calculated by the formula in Comparison 1 (Apply methods without considering network modules.). “NET” presents the type I error rates evaluated by the formula in Comparison 2 (Apply methods by considering network modules).

**Table E**. The estimated type I error rates of the six multiple phenotype association tests divided by the nominal significance level for 60, 80, and 100 mixture phenotypes (half continuous phenotypes and half binary phenotypes with balanced case-control ratios) under model 5. The type I error rates are evaluated using 500 MC runs (equivalent to $10^{6}$ replicates).

| **Mixture Phenotypes**  **Model 5** | | | **ceCLC** | | **CLC** | | **HCLC** | | **MultiPhen** | | **O’Brien** | | **Omnibus** | |
| --- | --- | --- | --- | --- | --- | --- | --- | --- | --- | --- | --- | --- | --- | --- |
| $K$ | Sample | $\alpha$-level | N.O. | NET | N.O. | NET | N.O. | NET | N.O. | NET | N.O. | NET | N.O. | NET |
| 60 | 2000 | 0.001 | 1.017 | 1.011 | 0.888 | 0.948 | 0.872 | 0.908 | **1.451** | **1.154** | 1.015 | 1.017 | 0.816 | 0.926 |
|  |  | 0.0001 | 0.780 | 1.060 | 0.780 | 1.000 | 0.770 | 1.040 | **1.580** | **1.240** | 1.060 | 0.890 | 0.750 | 0.890 |
|  | 4000 | 0.001 | 1.047 | 1.061 | 0.919 | 0.968 | 0.926 | 0.997 | **1.225** | **1.069** | 1.024 | 1.057 | 0.918 | 0.959 |
|  |  | 0.0001 | 0.980 | 1.060 | 0.900 | 0.950 | 0.930 | 1.070 | **1.350** | 1.150 | 1.180 | 1.180 | 0.840 | 0.880 |
| 80 | 2000 | 0.001 | 0.973 | 1.006 | 0.852 | 0.929 | 0.854 | 0.959 | **1.846** | **1.283** | 1.028 | 1.056 | 0.837 | 0.891 |
|  |  | 0.0001 | 0.970 | 0.840 | 0.930 | 1.050 | 0.750 | 0.940 | **2.080** | **1.220** | 1.110 | **1.370** | 0.640 | 0.720 |
|  | 4000 | 0.001 | 1.032 | 0.998 | 0.893 | 0.956 | 0.873 | 0.911 | **1.347** | **1.087** | 0.977 | 1.033 | 0.872 | 0.928 |
|  |  | 0.0001 | 1.100 | 0.950 | 0.930 | 0.970 | 0.860 | 0.850 | **1.250** | 1.070 | 1.090 | 1.120 | 0.850 | 0.930 |
| 100 | 2000 | 0.001 | 0.843 | 0.964 | 0.834 | 0.891 | 0.706 | 0.837 | **2.103** | **1.266** | 0.978 | 1.007 | 0.700 | 0.824 |
|  |  | 0.0001 | 0.790 | 0.980 | 0.740 | 0.760 | 0.560 | 0.890 | **2.350** | **1.360** | 1.050 | 1.100 | 0.540 | 0.760 |
|  | 4000 | 0.001 | 0.937 | 1.003 | 0.931 | 0.943 | 0.884 | 0.935 | **1.483** | **1.100** | 1.028 | 1.026 | 0.861 | 0.888 |
|  |  | 0.0001 | 0.880 | 0.990 | 0.860 | 1.020 | 0.660 | 0.900 | **1.590** | 1.110 | 0.970 | 1.020 | 0.690 | 0.850 |

*Notes:* bold-faced values indicate that the values are beyond the upper bounds of the 95% CIs. 95% CIs for type I error rates divided by nominal significance levels 0.001 and 0.0001 are (0.938, 1.062) and (0.804, 1.196), respectively. “N.O” represents the type I error rates calculated by the formula in Comparison 1 (Apply methods without considering network modules.). “NET” presents the type I error rates evaluated by the formula in Comparison 2 (Apply methods by considering network modules).

**Table F**. The estimated type I error rates of the six multiple phenotype association tests divided by the nominal significance level for 60, 80, and 100 mixture phenotypes (half continuous phenotypes and half binary phenotypes with balanced case-control ratios) under model 6. The type I error rates are evaluated using 500 MC runs (equivalent to $10^{6}$ replicates).

| **Mixture Phenotypes**  **Model 6** | | | **ceCLC** | | **CLC** | | **HCLC** | | **MultiPhen** | | **O’Brien** | | **Omnibus** | |
| --- | --- | --- | --- | --- | --- | --- | --- | --- | --- | --- | --- | --- | --- | --- |
| $K$ | Sample | $\alpha$-level | N.O. | NET | N.O. | NET | N.O. | NET | N.O. | NET | N.O. | NET | N.O. | NET |
| 60 | 2000 | 0.001 | 0.949 | 0.980 | 0.840 | 0.958 | 0.868 | 0.891 | **1.460** | **1.143** | 1.002 | **1.124** | 0.804 | 0.898 |
|  |  | 0.0001 | 0.960 | 1.020 | 0.830 | 1.000 | 0.830 | 0.940 | **1.660** | **1.250** | 0.900 | 1.030 | 0.770 | 0.850 |
|  | 4000 | 0.001 | 1.041 | 1.036 | 0.953 | 0.996 | 0.913 | 0.911 | **1.212** | 1.025 | 0.985 | **1.130** | 0.882 | 0.952 |
|  |  | 0.0001 | 0.990 | 0.980 | 0.950 | 1.120 | 0.780 | 0.870 | **1.220** | 1.100 | 1.030 | **1.320** | 0.820 | 0.940 |
| 80 | 2000 | 0.001 | 0.921 | 0.952 | 0.848 | 0.965 | 0.787 | 0.867 | **1.761** | **1.193** | 0.994 | **1.103** | 0.754 | 0.887 |
|  |  | 0.0001 | 0.800 | 0.890 | 0.780 | 0.800 | 0.630 | 0.870 | **1.840** | **1.310** | 0.940 | 1.050 | 0.540 | 0.730 |
|  | 4000 | 0.001 | 0.989 | 1.040 | 0.974 | 1.021 | 0.923 | 0.926 | **1.363** | 1.061 | 1.026 | **1.126** | 0.917 | 0.898 |
|  |  | 0.0001 | 0.820 | 0.950 | 0.880 | 0.950 | 0.770 | 0.930 | **1.420** | 1.130 | 1.040 | **1.360** | 0.900 | 0.920 |
| 100 | 2000 | 0.001 | 0.885 | 0.954 | 0.815 | 0.961 | 0.714 | 0.895 | **2.097** | **1.317** | 0.947 | 1.048 | 0.656 | 0.898 |
|  |  | 0.0001 | 0.780 | 1.060 | 0.710 | 0.940 | 0.700 | 1.020 | **2.460** | **1.400** | 0.960 | **1.260** | 0.640 | 0.880 |
|  | 4000 | 0.001 | 0.974 | 0.976 | 0.915 | 0.924 | 0.881 | 0.897 | **1.445** | **1.069** | 0.994 | **1.137** | 0.844 | 0.861 |
|  |  | 0.0001 | 0.980 | 1.160 | 0.810 | 0.960 | 0.920 | 0.900 | **1.800** | 1.040 | 0.910 | **1.480** | 0.940 | 0.970 |

*Notes:* bold-faced values indicate that the values are beyond the upper bounds of the 95% CIs. 95% CIs for type I error rates divided by nominal significance levels 0.001 and 0.0001 are (0.938, 1.062) and (0.804, 1.196), respectively. “N.O” represents the type I error rates calculated by the formula in Comparison 1 (Apply methods without considering network modules.). “NET” presents the type I error rates evaluated by the formula in Comparison 2 (Apply methods by considering network modules).

**Table G**. The estimated type I error rates of the six multiple phenotype association tests divided by the nominal significance level for 60, 80, and 100 binary phenotypes (with extremely unbalanced case-control ratios) under model 1. The type I error rates are evaluated using 500 MC runs (equivalent to $10^{6}$ replicates).

| **Binary Phenotypes**  **Model 1** | | | **ceCLC** | | **CLC** | | **HCLC** | | **MultiPhen** | | **O’Brien** | | **Omnibus** | |
| --- | --- | --- | --- | --- | --- | --- | --- | --- | --- | --- | --- | --- | --- | --- |
| $K$ | Sample | $\alpha$-level | N.O. | NET | N.O. | NET | N.O. | NET | N.O. | NET | N.O. | NET | N.O. | NET |
| 60 | 2000 | 0.001 | 0.965 | 0.973 | 0.657 | 0.715 | 0.792 | 0.811 | **3.865** | **2.433** | 0.960 | 1.014 | 0.550 | 0.531 |
|  |  | 0.0001 | 1.020 | 1.130 | 0.510 | 0.680 | 0.830 | 0.820 | **5.310** | **2.760** | 1.190 | **1.330** | 0.400 | 0.440 |
|  | 4000 | 0.001 | 1.027 | 1.011 | 0.748 | 0.846 | 0.871 | 0.852 | **2.251** | **1.695** | 0.988 | 1.013 | 0.766 | 0.747 |
|  |  | 0.0001 | 1.030 | 1.120 | 0.690 | 0.740 | 0.860 | 0.810 | **2.510** | **1.870** | 1.010 | 1.070 | 0.600 | 0.610 |
| 80 | 2000 | 0.001 | 0.969 | 1.011 | 0.659 | 0.682 | 0.747 | 0.775 | **5.638** | **2.972** | 0.924 | **1.051** | 0.550 | 0.530 |
|  |  | 0.0001 | 0.870 | 0.910 | 0.570 | 0.620 | 0.780 | 0.630 | **8.280** | **3.430** | 0.960 | 1.190 | 0.500 | 0.320 |
|  | 4000 | 0.001 | 1.000 | 1.035 | 0.790 | 0.778 | 0.900 | 0.846 | **2.820** | **1.964** | 0.969 | 0.975 | 0.785 | 0.761 |
|  |  | 0.0001 | **1.240** | 1.190 | 0.740 | 0.760 | 0.990 | 0.900 | **3.620** | **2.290** | 0.940 | 1.180 | 0.670 | 0.680 |
| 100 | 2000 | 0.001 | 0.965 | 1.053 | 0.663 | 0.702 | 0.819 | 0.826 | **8.393** | **3.867** | 0.926 | 1.016 | 0.553 | 0.580 |
|  |  | 0.0001 | 1.000 | 1.110 | 0.600 | 0.710 | 0.740 | 0.910 | **13.66** | **5.150** | 1.020 | 1.190 | 0.470 | 0.500 |
|  | 4000 | 0.001 | 1.034 | 1.061 | 0.730 | 0.790 | 0.847 | 0.866 | **3.454** | **2.212** | 0.971 | 1.014 | 0.681 | 0.728 |
|  |  | 0.0001 | 1.070 | 1.120 | 0.640 | 0.840 | 0.810 | 0.880 | **4.500** | **2.670** | **1.030** | **1.220** | 0.600 | 0.490 |

*Notes:* bold-faced values indicate that the values are beyond the upper bounds of the 95% CIs. 95% CIs for type I error rates divided by nominal significance levels 0.001 and 0.0001 are (0.938, 1.062) and (0.804, 1.196), respectively. “N.O” represents the type I error rates calculated by the formula in Comparison 1 (Apply methods without considering network modules.). “NET” presents the type I error rates evaluated by the formula in Comparison 2 (Apply methods by considering network modules).

**Table H**. The estimated type I error rates of the six multiple phenotype association tests divided by the nominal significance level for 60, 80, and 100 binary phenotypes (with extremely unbalanced case-control ratios) under model 2. The type I error rates are evaluated using 500 MC runs (equivalent to $10^{6}$ replicates).

| **Binary Phenotypes**  **Model 2** | | | **ceCLC** | | **CLC** | | **HCLC** | | **MultiPhen** | | **O’Brien** | | **Omnibus** | |
| --- | --- | --- | --- | --- | --- | --- | --- | --- | --- | --- | --- | --- | --- | --- |
| $K$ | Sample | $\alpha$-level | N.O. | NET | N.O. | NET | N.O. | NET | N.O. | NET | N.O. | NET | N.O. | NET |
| 60 | 2000 | 0.001 | 0.951 | 1.034 | 0.729 | 0.712 | 0.784 | 0.776 | **4.020** | **2.401** | 0.910 | **1.070** | 0.585 | 0.549 |
|  |  | 0.0001 | 0.900 | 1.100 | 0.590 | 0.730 | 0.670 | 0.830 | **5.190** | **2.630** | 0.800 | **1.420** | 0.420 | 0.440 |
|  | 4000 | 0.001 | 0.994 | 1.062 | 0.835 | 0.852 | 0.887 | 0.878 | **2.181** | **1.703** | 0.983 | 1.006 | 0.730 | 0.742 |
|  |  | 0.0001 | 1.170 | 1.100 | 0.720 | 0.950 | 0.910 | 0.910 | **2.640** | **1.890** | 0.920 | 1.130 | 0.520 | 0.630 |
| 80 | 2000 | 0.001 | 0.977 | 1.061 | 0.671 | 0.715 | 0.807 | 0.793 | **5.751** | **2.996** | 0.897 | **1.125** | 0.543 | 0.542 |
|  |  | 0.0001 | 1.130 | 1.170 | 0.570 | 0.690 | 0.870 | 0.880 | **8.540** | **3.650** | 1.050 | **1.440** | 0.430 | 0.370 |
|  | 4000 | 0.001 | 1.027 | 1.054 | 0.811 | 0.829 | 0.904 | 0.823 | **2.722** | **1.945** | 0.927 | 1.037 | 0.717 | 0.761 |
|  |  | 0.0001 | 1.170 | 1.090 | 0.610 | 0.940 | 0.920 | 0.850 | **3.440** | **2.330** | 1.090 | **1.300** | 0.750 | 0.520 |
| 100 | 2000 | 0.001 | 0.992 | 1.029 | 0.675 | 0.695 | 0.791 | 0.789 | **8.261** | **3.754** | 0.906 | 1.036 | 0.506 | 0.543 |
|  |  | 0.0001 | 1.000 | 1.180 | 0.600 | 0.630 | 0.810 | 0.930 | **12.77** | **4.530** | 0.930 | **1.370** | 0.490 | 0.490 |
|  | 4000 | 0.001 | 1.061 | 1.090 | 0.814 | 0.817 | 0.915 | 0.874 | **3.458** | **2.279** | 0.975 | 1.036 | 0.743 | 0.764 |
|  |  | 0.0001 | 1.120 | 1.100 | 0.860 | 0.750 | 0.980 | 0.970 | **4.380** | **2.730** | 1.040 | 1.170 | 0.610 | 0.750 |

*Notes:* bold-faced values indicate that the values are beyond the upper bounds of the 95% CIs. 95% CIs for type I error rates divided by nominal significance levels 0.001 and 0.0001 are (0.938, 1.062) and (0.804, 1.196), respectively. “N.O” represents the type I error rates calculated by the formula in Comparison 1 (Apply methods without considering network modules.). “NET” presents the type I error rates evaluated by the formula in Comparison 2 (Apply methods by considering network modules).

**Table I**. The estimated type I error rates of the six multiple phenotype association tests divided by the nominal significance level for 60, 80, and 100 binary phenotypes (with extremely unbalanced case-control ratios) under model 3. The type I error rates are evaluated using 500 MC runs (equivalent to $10^{6}$ replicates).

| **Binary Phenotypes**  **Model 3** | | | **ceCLC** | | **CLC** | | **HCLC** | | **MultiPhen** | | **O’Brien** | | **Omnibus** | |
| --- | --- | --- | --- | --- | --- | --- | --- | --- | --- | --- | --- | --- | --- | --- |
| $K$ | Sample | $\alpha$-level | N.O. | NET | N.O. | NET | N.O. | NET | N.O. | NET | N.O. | NET | N.O. | NET |
| 60 | 2000 | 0.001 | 0.949 | 1.003 | 0.704 | 0.730 | 0.789 | 0.761 | **3.525** | **2.089** | 0.870 | 1.054 | 0.571 | 0.578 |
|  |  | 0.0001 | 1.040 | 1.160 | 0.720 | 0.820 | 0.850 | 0.910 | **4.610** | **2.390** | 0.870 | 1.120 | 0.500 | 0.680 |
|  | 4000 | 0.001 | 0.970 | 1.040 | 0.786 | 0.905 | 0.935 | 0.903 | **2.076** | **1.494** | 0.969 | 1.014 | 0.786 | 0.727 |
|  |  | 0.0001 | 1.050 | 1.100 | 0.760 | 0.800 | 0.950 | 0.790 | **2.670** | **1.580** | 0.890 | **1.210** | 0.770 | 0.710 |
| 80 | 2000 | 0.001 | 0.940 | 1.044 | 0.632 | 0.689 | 0.773 | 0.855 | **4.629** | **2.299** | 0.855 | 1.056 | 0.519 | 0.544 |
|  |  | 0.0001 | 1.070 | 1.120 | 0.660 | 0.840 | 0.700 | 1.050 | **6.390** | **2.770** | 0.840 | 1.190 | 0.430 | 0.460 |
|  | 4000 | 0.001 | 1.009 | 1.061 | 0.770 | 0.850 | 0.925 | 0.899 | **2.358** | **1.617** | 0.954 | **1.093** | 0.728 | 0.715 |
|  |  | 0.0001 | 0.980 | 1.120 | 0.780 | 0.900 | 0.930 | 0.820 | **2.900** | **1.690** | 1.000 | 1.160 | 0.740 | 0.580 |
| 100 | 2000 | 0.001 | 0.968 | 1.050 | 0.680 | 0.701 | 0.731 | 0.811 | **6.697** | **3.000** | 0.876 | 1.058 | 0.533 | 0.570 |
|  |  | 0.0001 | 0.930 | 1.110 | 0.720 | 0.710 | 0.680 | 0.920 | **10.29** | **3.760** | 0.790 | 1.150 | 0.400 | 0.430 |
|  | 4000 | 0.001 | 0.998 | 1.060 | 0.764 | 0.794 | 0.898 | 0.873 | **2.978** | **1.831** | 0.987 | **1.093** | 0.690 | 0.715 |
|  |  | 0.0001 | 1.140 | 1.190 | 0.650 | 0.820 | 0.750 | 0.970 | **3.330** | **2.040** | 0.930 | 1.190 | 0.600 | 0.760 |

*Notes:* bold-faced values indicate that the values are beyond the upper bounds of the 95% CIs. 95% CIs for type I error rates divided by nominal significance levels 0.001 and 0.0001 are (0.938, 1.062) and (0.804, 1.196), respectively. “N.O” represents the type I error rates calculated by the formula in Comparison 1 (Apply methods without considering network modules.). “NET” presents the type I error rates evaluated by the formula in Comparison 2 (Apply methods by considering network modules).

**Table J**. The estimated type I error rates of the six multiple phenotype association tests divided by the nominal significance level for 60, 80, and 100 binary phenotypes (with extremely unbalanced case-control ratios) under model 4. The type I error rates are evaluated using 500 MC runs (equivalent to $10^{6}$ replicates).

| **Binary Phenotypes**  **Model 4** | | | **ceCLC** | | **CLC** | | **HCLC** | | **MultiPhen** | | **O’Brien** | | **Omnibus** | |
| --- | --- | --- | --- | --- | --- | --- | --- | --- | --- | --- | --- | --- | --- | --- |
| $K$ | Sample | $\alpha$-level | N.O. | NET | N.O. | NET | N.O. | NET | N.O. | NET | N.O. | NET | N.O. | NET |
| 60 | 2000 | 0.001 | 0.978 | 1.036 | 0.656 | 0.750 | 0.801 | 0.892 | **3.362** | **1.986** | 0.924 | 1.055 | 0.537 | 0.574 |
|  |  | 0.0001 | 1.110 | 1.120 | 0.700 | 0.710 | 0.720 | 0.830 | **4.040** | **2.430** | 1.050 | 1.440 | 0.340 | 0.470 |
|  | 4000 | 0.001 | 1.031 | 1.049 | 0.827 | 0.824 | 0.929 | 0.853 | **2.068** | **1.527** | 0.968 | 1.061 | 0.724 | 0.759 |
|  |  | 0.0001 | 1.080 | 1.100 | 0.800 | 0.790 | 1.050 | 0.800 | **2.390** | **1.670** | 1.180 | 1.170 | 0.650 | 0.600 |
| 80 | 2000 | 0.001 | 0.946 | 1.059 | 0.666 | 0.736 | 0.792 | 0.853 | **4.719** | **2.412** | 0.896 | **1.119** | 0.543 | 0.585 |
|  |  | 0.0001 | 1.050 | 1.110 | 0.620 | 0.810 | 0.860 | 0.850 | **6.410** | **2.780** | 0.830 | **1.420** | 0.420 | 0.500 |
|  | 4000 | 0.001 | 0.944 | 1.061 | 0.786 | 0.842 | 0.895 | 0.913 | **2.417** | **1.707** | 0.919 | 1.053 | 0.750 | 0.769 |
|  |  | 0.0001 | 1.070 | **1.250** | 0.630 | 0.860 | 0.830 | 0.880 | **3.170** | **1.930** | 0.960 | 1.120 | 0.740 | 0.800 |
| 100 | 2000 | 0.001 | 1.023 | 1.037 | 0.731 | 0.748 | 0.823 | 0.847 | **6.615** | **2.861** | 0.944 | 1.074 | 0.526 | 0.542 |
|  |  | 0.0001 | 1.090 | 1.170 | 0.740 | 0.780 | 0.730 | 1.040 | **10.04** | **3.580** | 0.950 | **1.230** | 0.430 | 0.470 |
|  | 4000 | 0.001 | 1.010 | 1.057 | 0.761 | 0.802 | 0.905 | 0.914 | **2.986** | **1.760** | 0.909 | 1.053 | 0.720 | 0.729 |
|  |  | 0.0001 | 0.960 | 1.120 | 0.660 | 0.590 | 0.790 | 0.930 | **3.770** | **1.930** | 0.900 | **1.290** | 0.680 | 0.650 |

*Notes:* bold-faced values indicate that the values are beyond the upper bounds of the 95% CIs. 95% CIs for type I error rates divided by nominal significance levels 0.001 and 0.0001 are (0.938, 1.062) and (0.804, 1.196), respectively. “N.O” represents the type I error rates calculated by the formula in Comparison 1 (Apply methods without considering network modules.). “NET” presents the type I error rates evaluated by the formula in Comparison 2 (Apply methods by considering network modules).

**Table K**. The estimated type I error rates of the six multiple phenotype association tests divided by the nominal significance level for 60, 80, and 100 binary phenotypes (with extremely unbalanced case-control ratios) under model 5. The type I error rates are evaluated using 500 MC runs (equivalent to $10^{6}$ replicates).

| **Binary Phenotypes**  **Model 5** | | | **ceCLC** | | **CLC** | | **HCLC** | | **MultiPhen** | | **O’Brien** | | **Omnibus** | |
| --- | --- | --- | --- | --- | --- | --- | --- | --- | --- | --- | --- | --- | --- | --- |
| $K$ | Sample | $\alpha$-level | N.O. | NET | N.O. | NET | N.O. | NET | N.O. | NET | N.O. | NET | N.O. | NET |
| 60 | 2000 | 0.001 | 0.974 | 1.044 | 0.727 | 0.715 | 0.866 | 0.869 | **3.530** | **2.140** | 0.973 | 1.058 | 0.560 | 0.541 |
|  |  | 0.0001 | 1.020 | 0.960 | 0.630 | 0.620 | 0.830 | 0.780 | **4.460** | **2.350** | 0.930 | 1.180 | 0.330 | 0.380 |
|  | 4000 | 0.001 | 1.025 | 1.056 | 0.804 | 0.837 | 0.883 | 0.899 | **2.053** | **1.540** | 0.944 | 1.062 | 0.734 | 0.746 |
|  |  | 0.0001 | 1.160 | 1.140 | 0.670 | 0.760 | 0.980 | 0.860 | **2.570** | **1.510** | 1.030 | 1.180 | 0.730 | 0.470 |
| 80 | 2000 | 0.001 | 0.965 | 1.057 | 0.687 | 0.740 | 0.807 | 0.875 | **4.909** | **2.588** | 0.909 | **1.130** | 0.522 | 0.577 |
|  |  | 0.0001 | 1.040 | 1.110 | 0.570 | 0.680 | 0.870 | 0.950 | **6.400** | **3.210** | 0.820 | **1.250** | 0.410 | 0.450 |
|  | 4000 | 0.001 | 0.993 | 1.036 | 0.838 | 0.819 | 0.879 | 0.882 | **2.450** | **1.732** | 0.924 | 1.056 | 0.713 | 0.722 |
|  |  | 0.0001 | 1.120 | 1.150 | 0.610 | 0.930 | 1.070 | 0.990 | **3.130** | **2.070** | 1.000 | **1.350** | 0.600 | 0.680 |
| 100 | 2000 | 0.001 | 0.871 | 1.060 | 0.714 | 0.736 | 0.746 | 0.807 | **6.884** | **3.209** | 0.874 | 1.058 | 0.550 | 0.537 |
|  |  | 0.0001 | 1.040 | 1.180 | 0.670 | 0.690 | 0.600 | 0.990 | **10.67** | **3.870** | 0.880 | 1.130 | 0.360 | 0.460 |
|  | 4000 | 0.001 | 0.969 | 1.099 | 0.819 | 0.804 | 0.915 | 0.897 | **3.179** | **1.904** | 0.914 | 1.050 | 0.786 | 0.733 |
|  |  | 0.0001 | 1.040 | 1.160 | 0.710 | 0.970 | 0.770 | 1.030 | **4.410** | **2.220** | 1.070 | 1.160 | 0.550 | 0.600 |

*Notes:* bold-faced values indicate that the values are beyond the upper bounds of the 95% CIs. 95% CIs for type I error rates divided by nominal significance levels 0.001 and 0.0001 are (0.938, 1.062) and (0.804, 1.196), respectively. “N.O” represents the type I error rates calculated by the formula in Comparison 1 (Apply methods without considering network modules.). “NET” presents the type I error rates evaluated by the formula in Comparison 2 (Apply methods by considering network modules).

**Table L**. The estimated type I error rates of the six multiple phenotype association tests divided by the nominal significance level for 60, 80, and 100 binary phenotypes (with extremely unbalanced case-control ratios) under model 6. The type I error rates are evaluated using 500 MC runs (equivalent to $10^{6}$ replicates).

| **Binary Phenotypes**  **Model 6** | | | **ceCLC** | | **CLC** | | **HCLC** | | **MultiPhen** | | **O’Brien** | | **Omnibus** | |
| --- | --- | --- | --- | --- | --- | --- | --- | --- | --- | --- | --- | --- | --- | --- |
| $K$ | Sample | $\alpha$-level | N.O. | NET | N.O. | NET | N.O. | NET | N.O. | NET | N.O. | NET | N.O. | NET |
| 60 | 2000 | 0.001 | 0.995 | 1.061 | 0.719 | 0.852 | 0.838 | 0.900 | **3.288** | **1.771** | 0.907 | 1.024 | 0.566 | 0.537 |
|  |  | 0.0001 | 1.130 | 1.180 | 0.710 | 0.780 | 0.800 | 1.010 | **4.290** | **2.030** | 1.000 | 1.140 | 0.410 | 0.470 |
|  | 4000 | 0.001 | 1.004 | 1.006 | 0.849 | 0.943 | 0.890 | 0.964 | **1.949** | **1.452** | 0.967 | **1.082** | 0.726 | 0.739 |
|  |  | 0.0001 | 1.120 | 1.130 | 0.770 | 1.020 | 1.070 | 1.120 | **2.200** | **1.580** | 1.070 | **1.280** | 0.710 | 0.850 |
| 80 | 2000 | 0.001 | 0.940 | 1.072 | 0.708 | 0.785 | 0.776 | 0.876 | **4.481** | **2.167** | 0.953 | 1.061 | 0.570 | 0.549 |
|  |  | 0.0001 | 0.980 | 1.070 | 0.690 | 0.810 | 0.740 | 0.880 | **6.110** | **2.520** | 0.840 | 1.130 | 0.420 | 0.400 |
|  | 4000 | 0.001 | 1.011 | **1.129** | 0.774 | 0.838 | 0.913 | 0.923 | **2.303** | **1.549** | 0.988 | **1.150** | 0.718 | 0.743 |
|  |  | 0.0001 | 0.910 | **1.240** | 0.670 | 1.120 | 0.810 | 0.990 | **3.130** | **1.910** | 0.900 | **1.360** | 0.770 | 0.770 |
| 100 | 2000 | 0.001 | 0.938 | 1.081 | 0.684 | 0.744 | 0.776 | 0.892 | **6.011** | **2.547** | 0.927 | 1.012 | 0.499 | 0.534 |
|  |  | 0.0001 | 0.890 | 1.160 | 0.600 | 0.810 | 0.590 | 1.040 | **8.730** | **3.130** | 0.940 | 1.120 | 0.390 | 0.410 |
|  | 4000 | 0.001 | 0.974 | 1.026 | 0.783 | 0.862 | 0.889 | 0.951 | **2.780** | **1.584** | 0.997 | 1.033 | 0.721 | 0.723 |
|  |  | 0.0001 | 0.890 | 1.180 | 0.680 | 1.010 | 0.900 | 1.080 | **3.560** | **1.990** | 0.910 | 1.110 | 0.680 | 0.660 |

*Notes:* bold-faced values indicate that the values are beyond the upper bounds of the 95% CIs. 95% CIs for type I error rates divided by nominal significance levels 0.001 and 0.0001 are (0.938, 1.062) and (0.804, 1.196), respectively. “N.O” represents the type I error rates calculated by the formula in Comparison 1 (Apply methods without considering network modules.). “NET” presents the type I error rates evaluated by the formula in Comparison 2 (Apply methods by considering network modules).

**Table M**. The estimated type I error rates of the six multiple phenotype association tests divided by the nominal significance level for 60, 80, and 100 mixture phenotypes (half continuous phenotypes and half binary phenotypes with balanced case-control ratios) under the null simulation model (no clusters/categories of phenotypes). The type I error rates are evaluated using 500 MC runs (equivalent to $10^{6}$ replicates).

| **Mixture Phenotypes**  **No cluster model** | | | **ceCLC** | | **CLC** | | **HCLC** | | **MultiPhen** | | **O’Brien** | | **Omnibus** | |
| --- | --- | --- | --- | --- | --- | --- | --- | --- | --- | --- | --- | --- | --- | --- |
| $K$ | Sample | $\alpha$-level | N.O. | NET | N.O. | NET | N.O. | NET | N.O. | NET | N.O. | NET | N.O. | NET |
| 60 | 2000 | 0.001 | 1.042 | 1.111 | 1.008 | 1.030 | 1.012 | 1.080 | **1.244** | 1.146 | 0.950 | 1.064 | 0.980 | 1.028 |
|  |  | 0.0001 | 1.174 | 1.090 | 0.970 | 1.140 | 0.980 | 1.174 | **1.380** | **1.270** | 1.052 | 1.060 | 0.880 | 1.163 |
|  | 4000 | 0.001 | 1.024 | 1.116 | 0.948 | 0.922 | 0.976 | 0.962 | **1.120** | 0.922 | 0.970 | 1.052 | 0.952 | 0.862 |
|  |  | 0.0001 | **1.240** | 1.120 | 0.996 | 0.854 | 0.787 | 0.950 | 1.090 | 0.940 | 0.860 | 1.122 | 0.890 | 1.002 |
| 80 | 2000 | 0.001 | 1.024 | 1.014 | 1.010 | 0.950 | 0.984 | 1.056 | **1.396** | **1.098** | 1.046 | **1.082** | 0.968 | 1.004 |
|  |  | 0.0001 | 1.160 | 1.063 | 1.000 | 0.999 | 1.050 | 1.140 | **1.732** | **1.280** | 1.080 | 1.141 | 0.970 | 1.030 |
|  | 4000 | 0.001 | **1.088** | 1.061 | 0.934 | 1.054 | 0.994 | 1.036 | **1.104** | 1.024 | 1.004 | 1.062 | 0.936 | 1.008 |
|  |  | 0.0001 | 1.120 | 1.073 | 0.942 | 1.043 | 0.995 | 1.152 | **1.300** | 1.120 | 1.140 | 1.154 | 0.957 | 1.010 |
| 100 | 2000 | 0.001 | 1.058 | 1.160 | 1.074 | 1.002 | 0.992 | 1.048 | **1.626** | 1.176 | 1.070 | 1.126 | 0.992 | 1.000 |
|  |  | 0.0001 | 1.020 | 1.018 | 1.070 | 1.050 | 0.776 | 1.123 | **1.750** | 1.160 | 0.940 | 1.120 | 0.750 | 0.830 |
|  | 4000 | 0.001 | **1.080** | 1.038 | 1.038 | 1.089 | 0.942 | 0.953 | **1.486** | **1.253** | 0.982 | 1.159 | 0.898 | 0.929 |
|  |  | 0.0001 | 1.107 | 1.162 | 1.085 | 1.018 | 1.109 | 1.055 | **1.529** | **1.286** | 1.074 | 1.162 | 0.900 | 0.969 |

*Notes:* bold-faced values indicate that the values are beyond the upper bounds of the 95% CIs. 95% CIs for type I error rates divided by nominal significance levels 0.001 and 0.0001 are (0.938, 1.062) and (0.804, 1.196), respectively. “N.O” represents the type I error rates calculated by the formula in Comparison 1 (Apply methods without considering network modules.). “NET” presents the type I error rates evaluated by the formula in Comparison 2 (Apply methods by considering network modules).

**Table N.** 33 unique SNPs identified by ceCLC for testing the association in NET (one SNP) or in N.O. (32 SNPs).

| **SNP** | **Position** | **Mapped gene** | **P value** | **Reported diseases** | **Reference** |
| --- | --- | --- | --- | --- | --- |
| **rs4148866^*^** | chr12: 123425575 | *ABCB9* | 2.97E-08 | - | - |
| rs13107325 | chr4:102267552 | *SLC39A8* | 4.60E-10 | M19.9 / M25.5 / M75.1 | [13-15] |
| **rs34333163** | chr4:102361960 | *SLC39A8* | 2.84E-08 | M19.9 / M25.5 / M75.1 | [13-15] |
| rs9468413 | chr6:28721895 | *-* | 2.91E-08 | - | - |
| rs880638 | chr6:28739135 | *-* | 4.02E-08 | - | - |
| rs9257802 | chr6:29375578 | *OR5V1* | 1.50E-08 | - | - |
| rs1264362 | chr6:30808813 | *HCG20* | 1.54E-09 | M07.3 | [16] |
| rs915664 | chr6:30826840 | *LINC00243* | 1.02E-08 | - | - |
| rs1264344 | chr6:30832800 | *LINC00243* | 9.31E-09 | - | - |
| rs1632854 | chr6:31007872 | *MUC21 / MUC22* | 2.77E-08 | M07.3 / M32.9 / M85.8 | [16-18] |
| rs4713422 | chr6:31032125 | *MUC22* | 8.63E-14 | M07.3 / M85.8 | [16, 18] |
| rs10947121 | chr6:31032220 | *MUC22* | 1.08E-13 | M07.3 / M85.8 | [16, 18] |
| rs2233967 | chr6:31113051 | *C6orf15 / PSORS1C1* | 4.61E-09 | M31.4 / M34 / M35.2 | [19-21] |
| rs1265086 | chr6:31142105 | *PSORS1C1 / PSORS1C2* | 5.07E-12 | M31.4 / M34 / M35.2 | [19-21] |
| rs130071 | chr6:31148433 | *PSORS1C1 / POU5F1* | 2.46E-10 | M07.3 / M31.4 / M34 / M35.2 | [16, 19-21] |
| rs4516988 | chr6:31208825 | *HCG27* | 7.30E-10 | M32.9 | [22] |
| rs4351302 | chr6:31209144 | *HCG27* | 4.08E-10 | M32.9 | [22] |
| rs9295967 | chr6:31216243 | *HCG27* | 6.65E-10 | M32.9 | [22] |
| rs9264733 | chr6:31276437 | *HLA-C / LINC02571* | 1.10E-08 | M07.3 / M31.4 | [16, 20] |
| rs3094682 | chr6:31296684 | *LINC02571* | 2.58E-09 | M07.3 | [16] |
| rs2596472 | chr6:31461190 | *HCP5 / MICB* | 1.33E-10 | M33.2 / M60 / M62.9 | [23-25] |
| rs3130615 | chr6:31507636 | *MICB* | 1.79E-11 | M60 | [24] |
| rs3132468 | chr6:31507709 | *MICB* | 1.57E-11 | M60 | [24] |
| rs3131635 | chr6:31508357 | *MICB* | 1.13E-11 | M60 | [24] |
| rs1065076 | chr6:31509904 | *MICB* | 1.26E-11 | M60 | [24] |
| rs2395045 | chr6:31516740 | *MICB* | 1.07E-09 | M60 | [24] |
| rs3093999 | chr6:31516773 | *MICB* | 8.32E-10 | M60 | [24] |
| rs3131631 | chr6:31516906 | *MICB* | 9.63E-10 | M60 | [24] |
| rs2734574 | chr6:31526111 | *MICB* | 5.30E-09 | M60 | [24] |
| **rs6916921** | chr6:31552649 | *ATP6V1G2 / DDX39B / LTA* | 2.28E-11 | M30.3 / M60 | [24, 26] |
| rs915895 | chr6:32222440 | *NOTCH4* | 1.94E-09 | M06.9 / M07.3 / M31.4 / M32.9 / M34 / M62.9 | [16, 20, 22, 25, 27] |
| rs915894 | chr6:32222613 | *NOTCH4* | 2.14E-08 | M06.9 / M07.3 / M31.4 / M32.9 / M34 / M62.9 | [16, 20, 22, 25, 27] |
| rs443198 | chr6:32222629 | *NOTCH4* | 1.73E-11 | M06.9 / M07.3 / M31.4 / M32.9 / M34 / M62.9 | [16, 20, 22, 25, 27] |

*Notes:* “^*^” indicates the unique SNP identified by ceCLC in NET. Bold-faced SNPs are the lead SNPs in the colocalization analysis. Mapped gene denotes the gene that includes the corresponding SNP with a 20kb window region. P-value is calculated by ceCLC. The corresponding diseases with ICD-10 codes in reported diseases are listed in Table S14.

**Table O**. ICD-10 codes and names of the14 reported diseases shown in Table N.

| **ICD-10** | **Disease** | **ICD-10** | **Disease** |
| --- | --- | --- | --- |
| M06.9 | rheumatoid arthritis | M33.2 | Polymyositis |
| M07.3 | psoriatic arthritis | M34 | systemic sclerosis |
| M19.9 | osteoarthritis | M35.2 | Behcet's disease |
| M25.5 | multisite chronic pain | M60 | myositis |
| M30.3 | Kawasaki disease | M62.9 | appendicular lean mass |
| M31.4 | Takayasu arteritis | M75.1 | rotator cuff syndrome |
| M32.9 | systemic lupus erythematosus | M85.8 | disorders of bone density and structure |

**Table P**. Simulation settings for the six models with $\hat{\lambda}_{1}=\beta\left(1,\cdots,1\right)^{T}$ and $\hat{\lambda}_{2}=\frac{2\beta}{k+1}\left(1,\cdots,k\right)^{T}$.

|  | Category | SNP 1-50 | SNP 51-100 | SNP 101-150 | SNP 151-200 |
| --- | --- | --- | --- | --- | --- |
| **Model 1** | 1 | $\hat{\lambda}_{1}$ | 0 | 0 | 0 |
|  | 2 | 0 | $-\hat{\lambda}_{1}$ | 0 | 0 |
| **Model 2** | 1 | $\hat{\lambda}_{1}$ | 0 | 0 | 0 |
|  | 2 | 0 | $-\hat{\lambda}_{2}$ | 0 | 0 |
| **Model 3** | 1 | $\hat{\lambda}_{1}$ | 0 | 0 | 0 |
|  | 2 | 0 | $-\hat{\lambda}_{1}$ | 0 | 0 |
|  | 3-5 | 0 | 0 | 0 | 0 |
| **Model 4** | 1 | $\hat{\lambda}_{1}$ | 0 | 0 | 0 |
|  | 2 | 0 | $-\hat{\lambda}_{2}$ | 0 | 0 |
|  | 3-5 | 0 | 0 | 0 | 0 |
| **Model 5** | 1 | $\hat{\lambda}_{1}$ | 0 | 0 | 0 |
|  | 2 | 0 | $-\hat{\lambda}_{1}$ | 0 | 0 |
|  | 3 | 0 | 0 | $\hat{\lambda}_{2}$ | 0 |
|  | 4 | 0 | 0 | 0 | $-\hat{\lambda}_{2}$ |
| **Model 6** | 1 | $\hat{\lambda}_{1}$ | 0 | 0 | 0 |
|  | 2 | 0 | $-\hat{\lambda}_{1}$ | 0 | 0 |
|  | 3 | 0 | 0 | $\hat{\lambda}_{2}$ | 0 |
|  | 4 | 0 | 0 | 0 | $-\hat{\lambda}_{2}$ |
|  | 5-10 | 0 | 0 | 0 | 0 |

**Fig A**. Power comparisons of the six tests as a function of effect size $\beta$ under six models. The number of mixture phenotypes (half continuous phenotypes and half binary phenotypes with balanced case-control ratios) is 80 and the sample size is 2,000. The power of all of the six tests is evaluated using 10 MC runs.

**
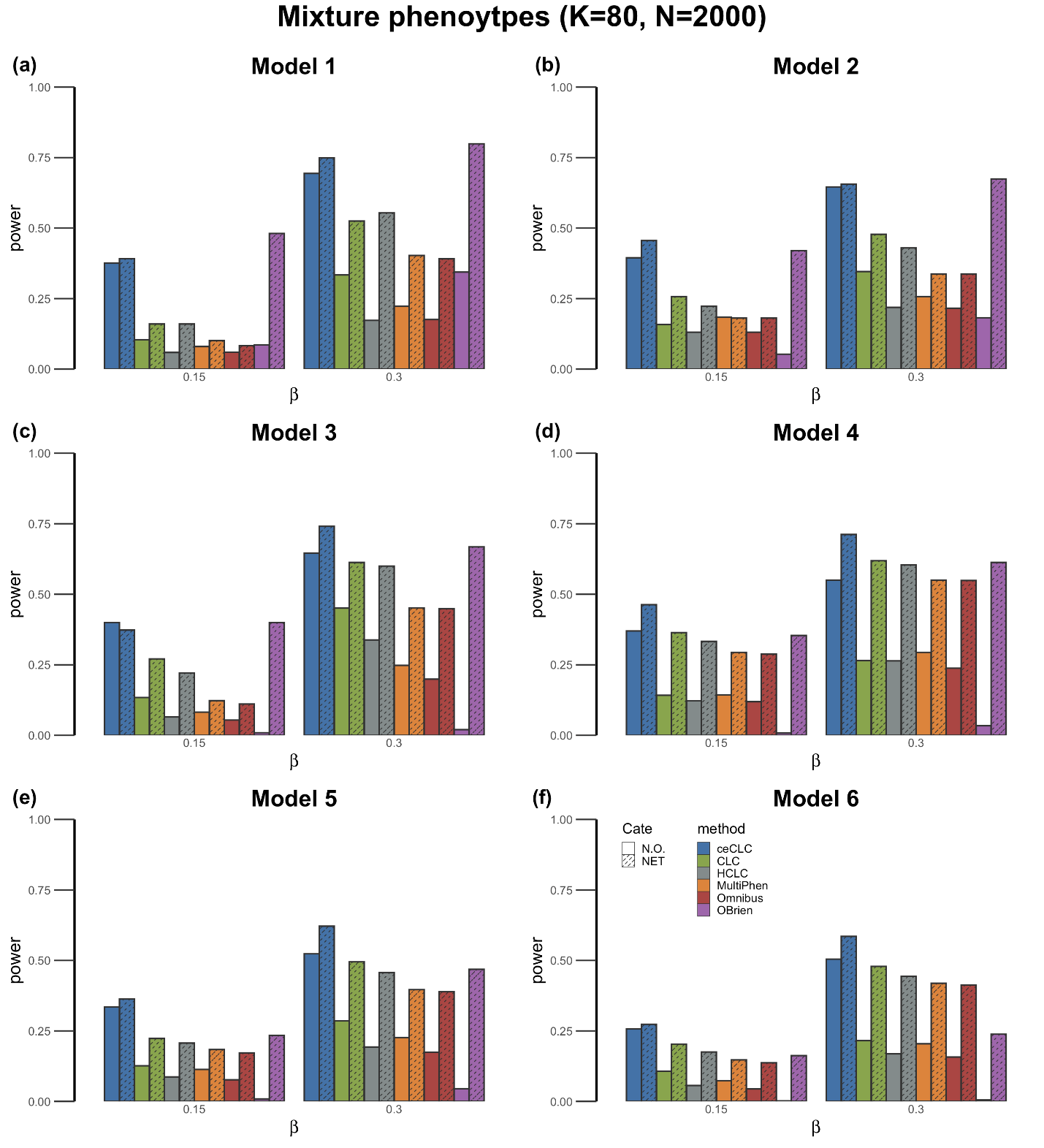
**

**Fig B**. Power comparisons of the six tests as a function of effect size $\beta$ under six models. The number of mixture phenotypes (half continuous phenotypes and half binary phenotypes with balanced case-control ratios) is 60 and the sample size is 2,000. The power of all of the six tests is evaluated using 10 MC runs.

**
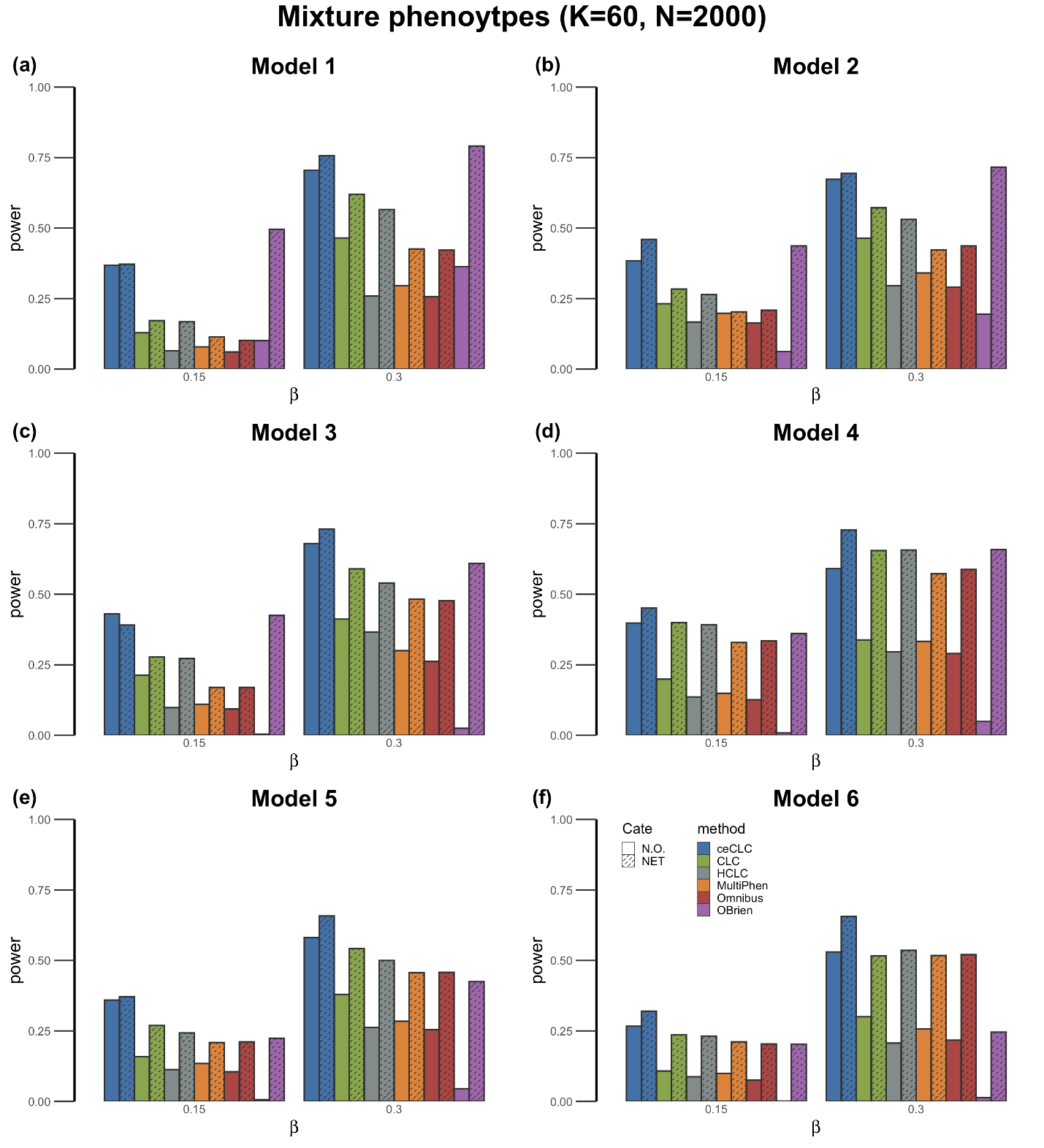
**

**Fig C**. Power comparisons of the six tests as a function of effect size $\beta$ under six models. The number of mixture phenotypes (half continuous phenotypes and half binary phenotypes with balanced case-control ratios) is 60 and the sample size is 4,000. The power of all of the six tests is evaluated using 10 MC runs.

**
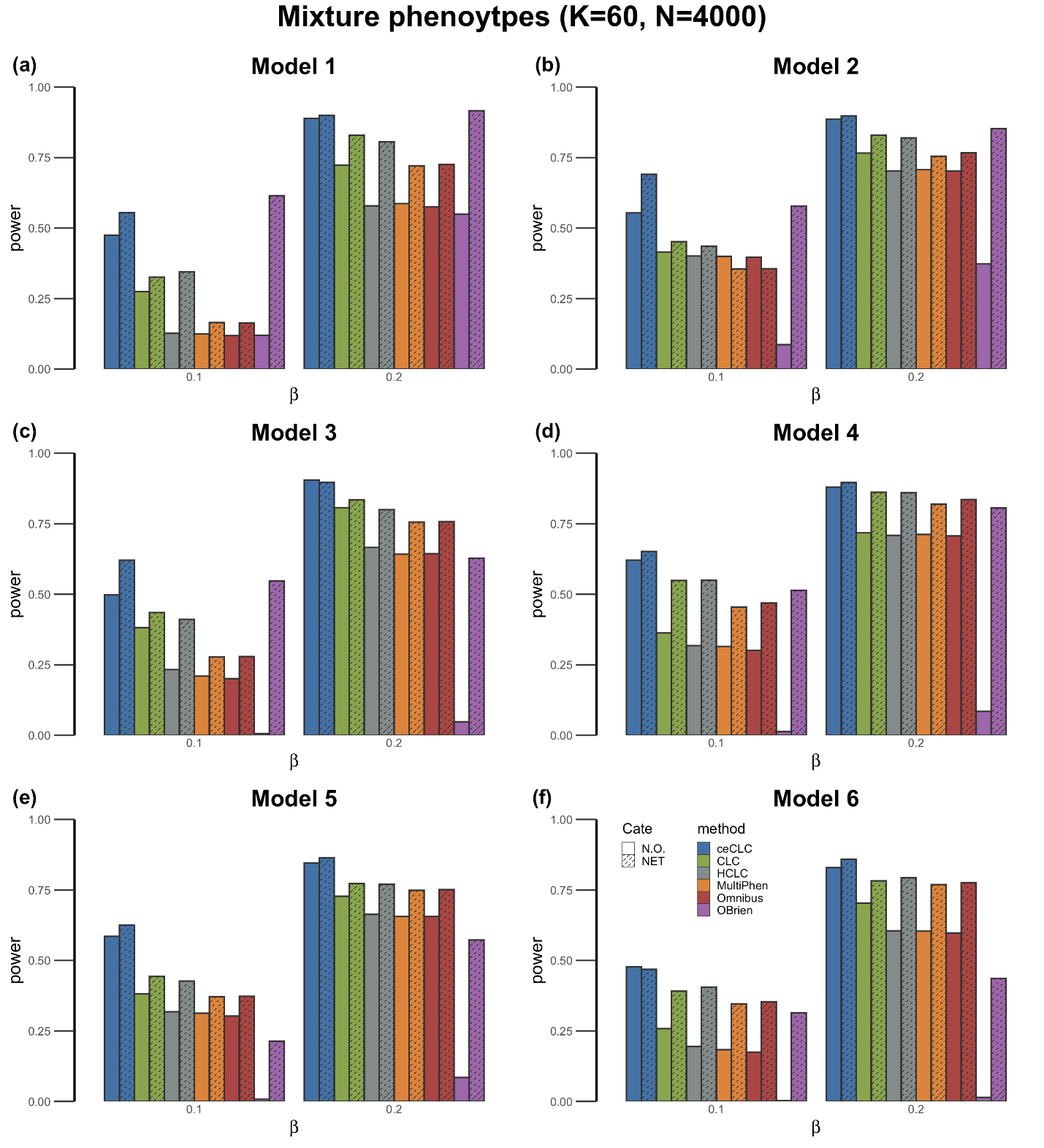
**

**Fig D**. Power comparisons of the six tests as a function of effect size $\beta$ under six models. The number of mixture phenotypes (half continuous phenotypes and half binary phenotypes with balanced case-control ratios) is 100 and the sample size is 2,000. The power of all of the six tests is evaluated using 10 MC runs.

**
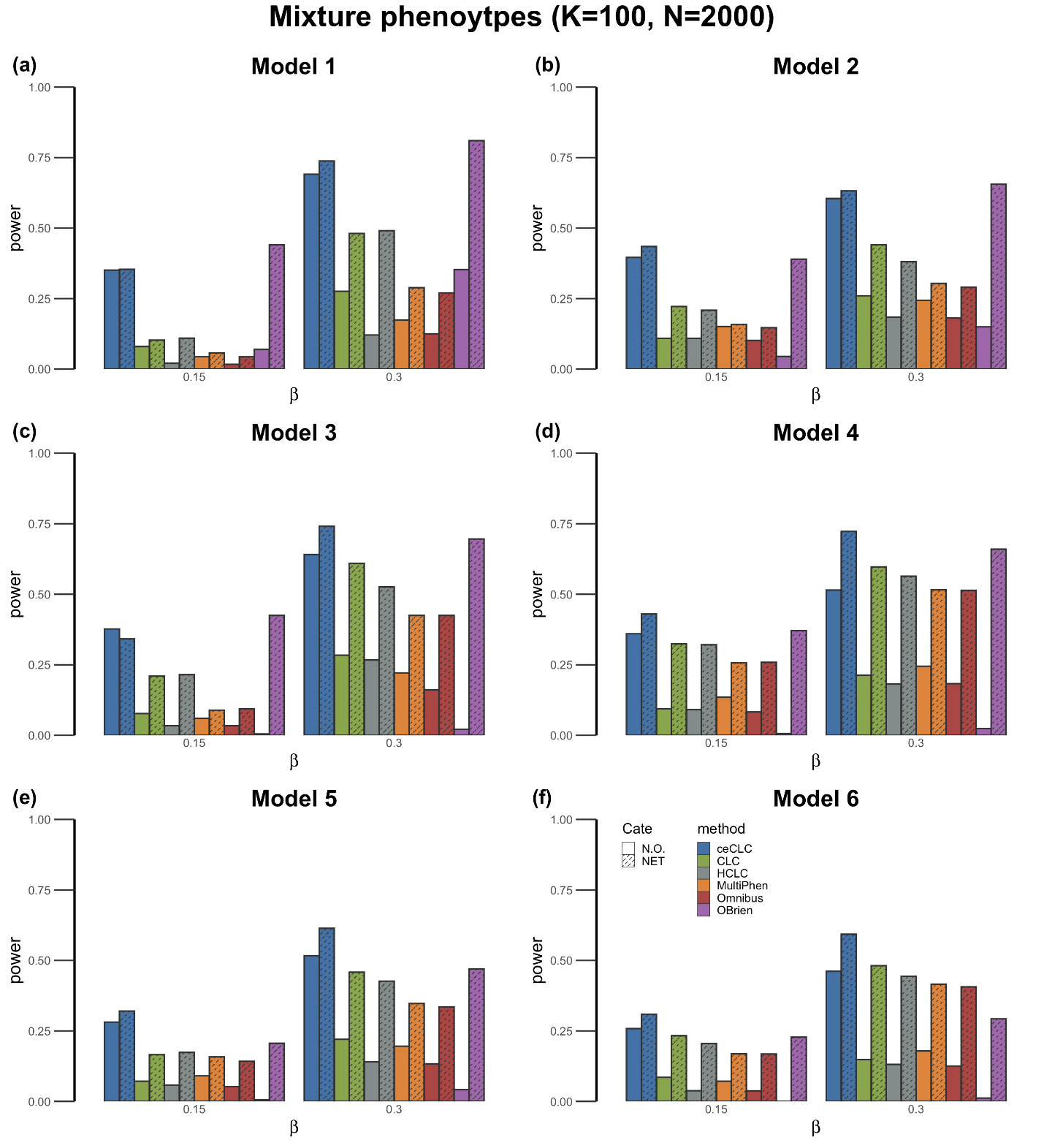
**

**Fig E**. Power comparisons of the six tests as a function of effect size $\beta$ under six models. The number of mixture phenotypes (half continuous phenotypes and half binary phenotypes with balanced case-control ratios) is 100 and the sample size is 4,000. The power of all of the six tests is evaluated using 10 MC runs.

**
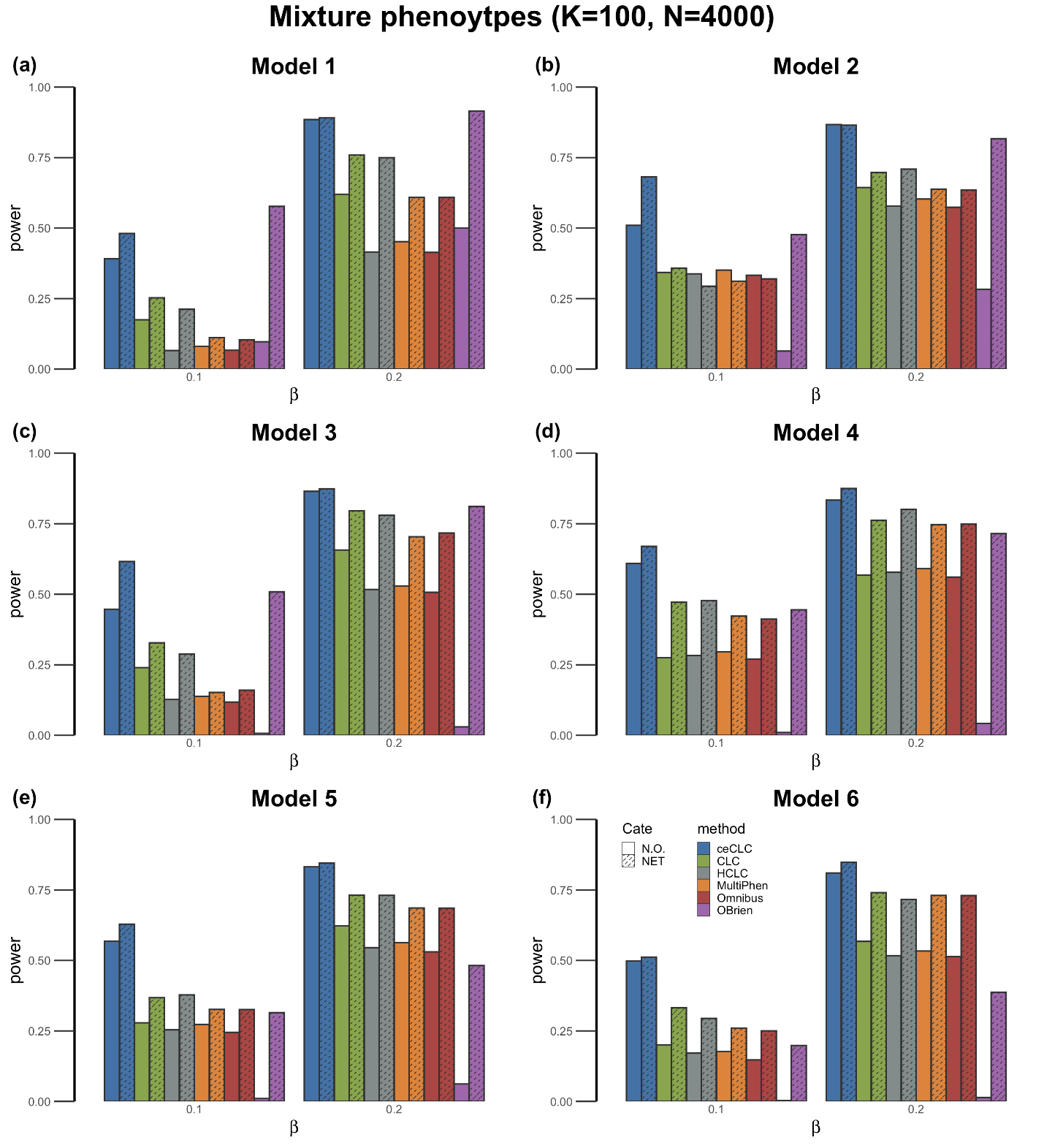
**

**Fig F**. Power comparisons of the six tests as a function of effect size $\beta$ under the six models. The number of binary phenotypes (with extremely unbalanced case-control ratios) is 80 and the sample size is 20,000. The power of all of the six tests is evaluated using 10 MC runs.

**
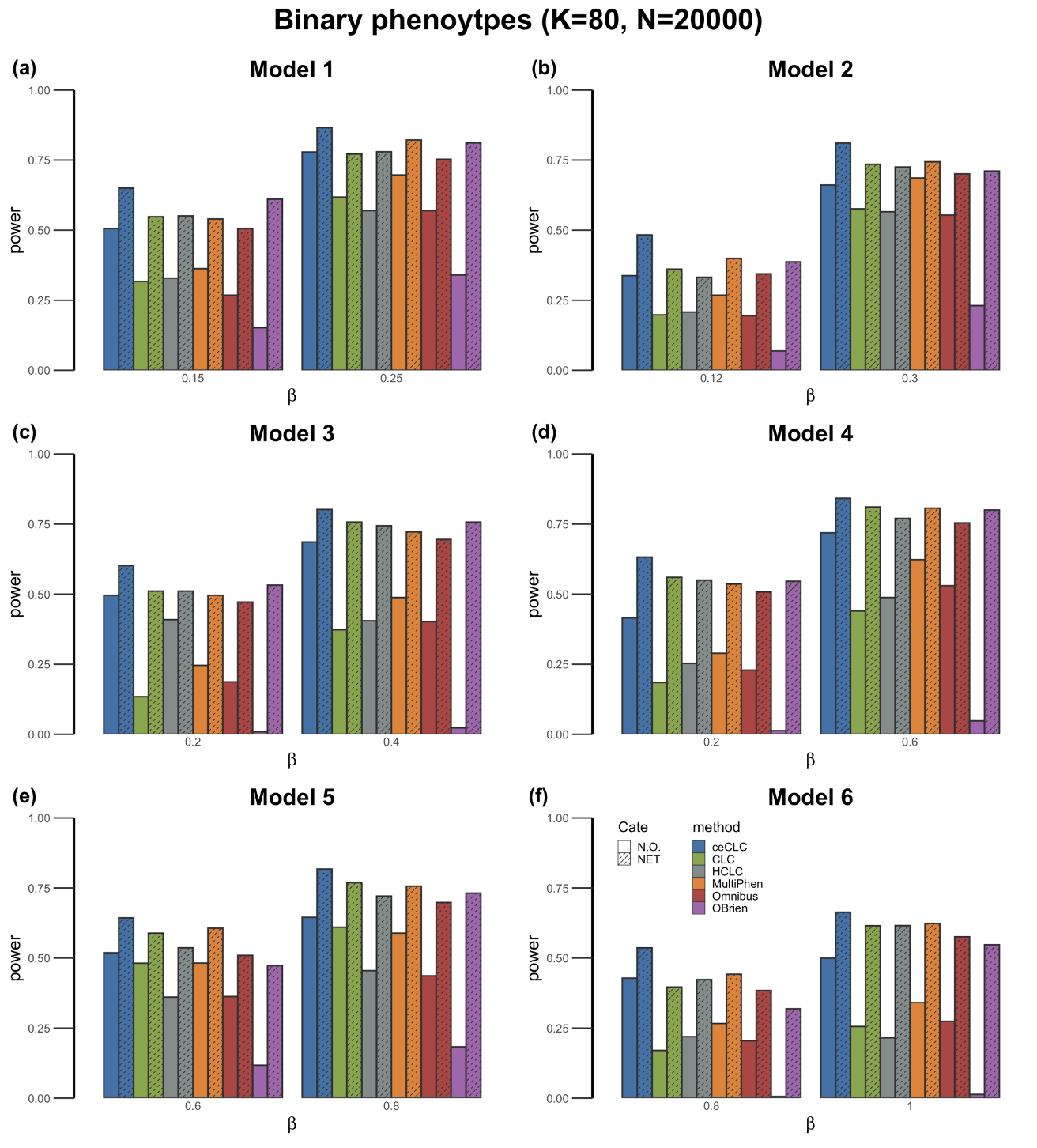
**

**Fig G**. Power comparisons of the six tests as a function of effect size $\beta$ under six models. The number of binary phenotypes (with extremely unbalanced case-control ratios) is 80 and the sample size is 10,000. The power of all of the six tests is evaluated using 10 MC runs.

**
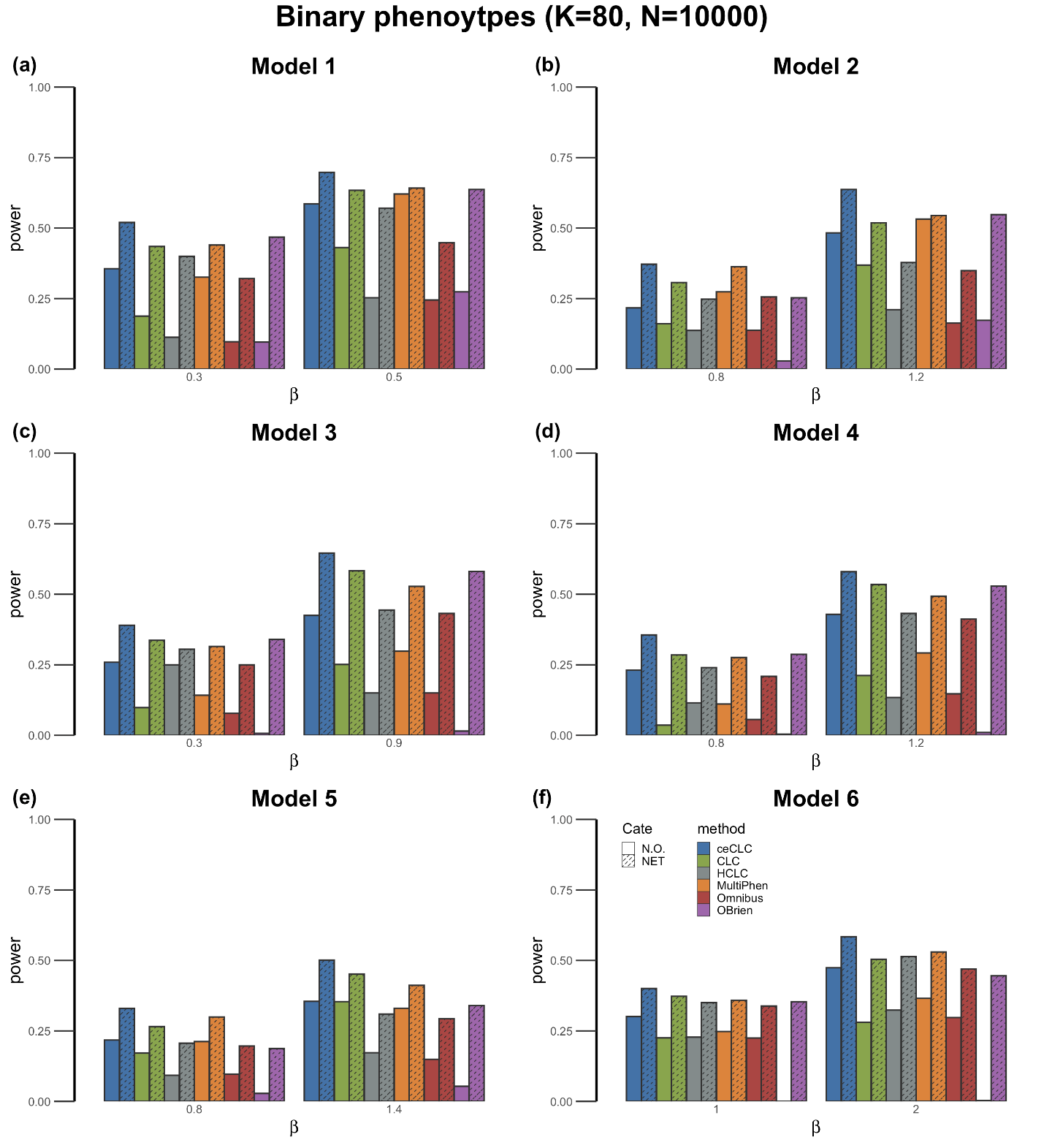
**

**Fig H**. Power comparisons of the six tests as a function of effect size $\beta$ under six models. The number of binary phenotypes (with extremely unbalanced case-control ratios) is 60 and the sample size is 10,000. The power of all of the six tests is evaluated using 10 MC runs.

**
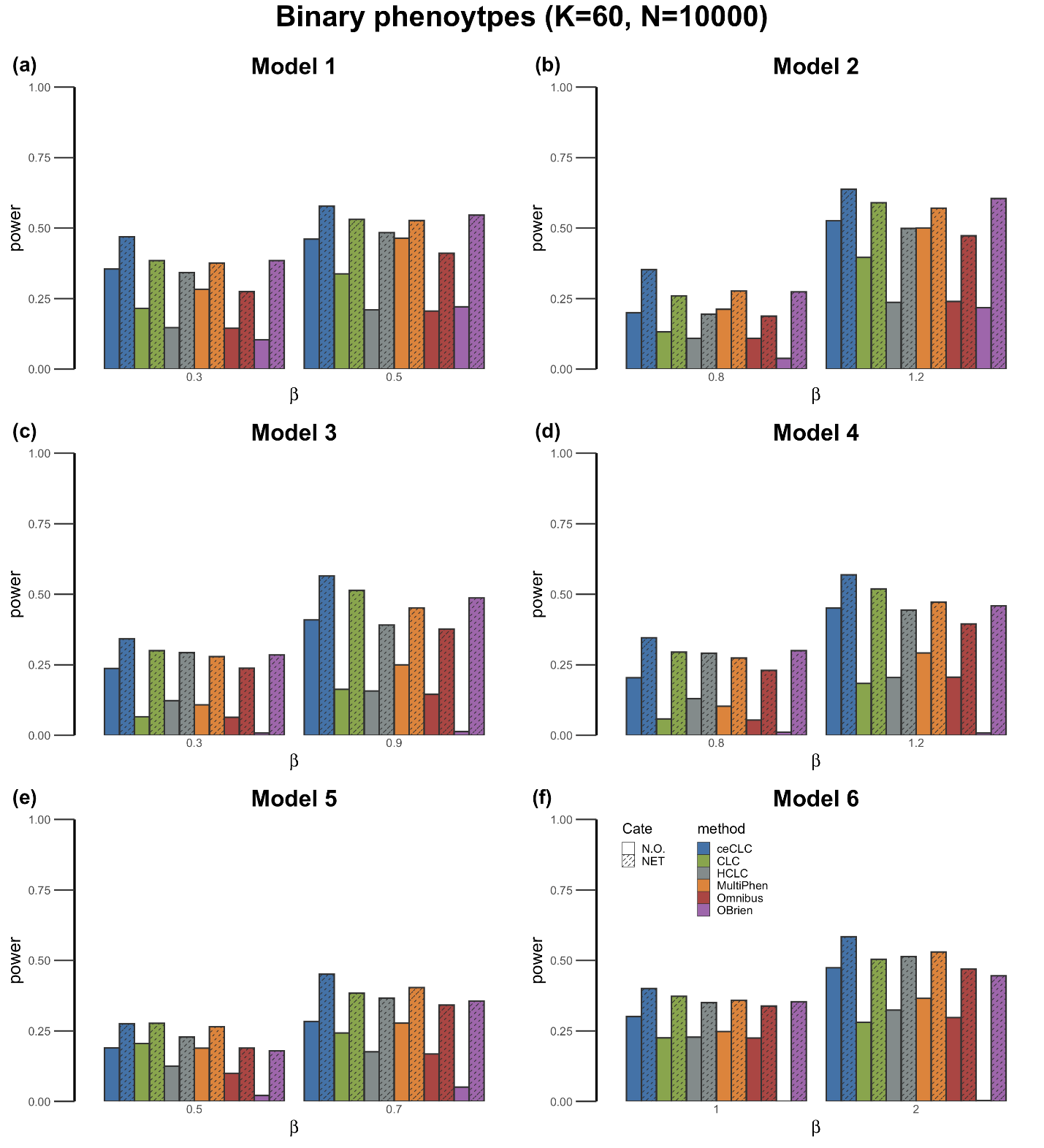
**

**Fig I**. Power comparisons of the six tests as a function of effect size $\beta$ under six models. The number of binary phenotypes (with extremely unbalanced case-control ratios) is 60 and the sample size is 20,000. The power of all of the six tests is evaluated using 10 MC runs.

**
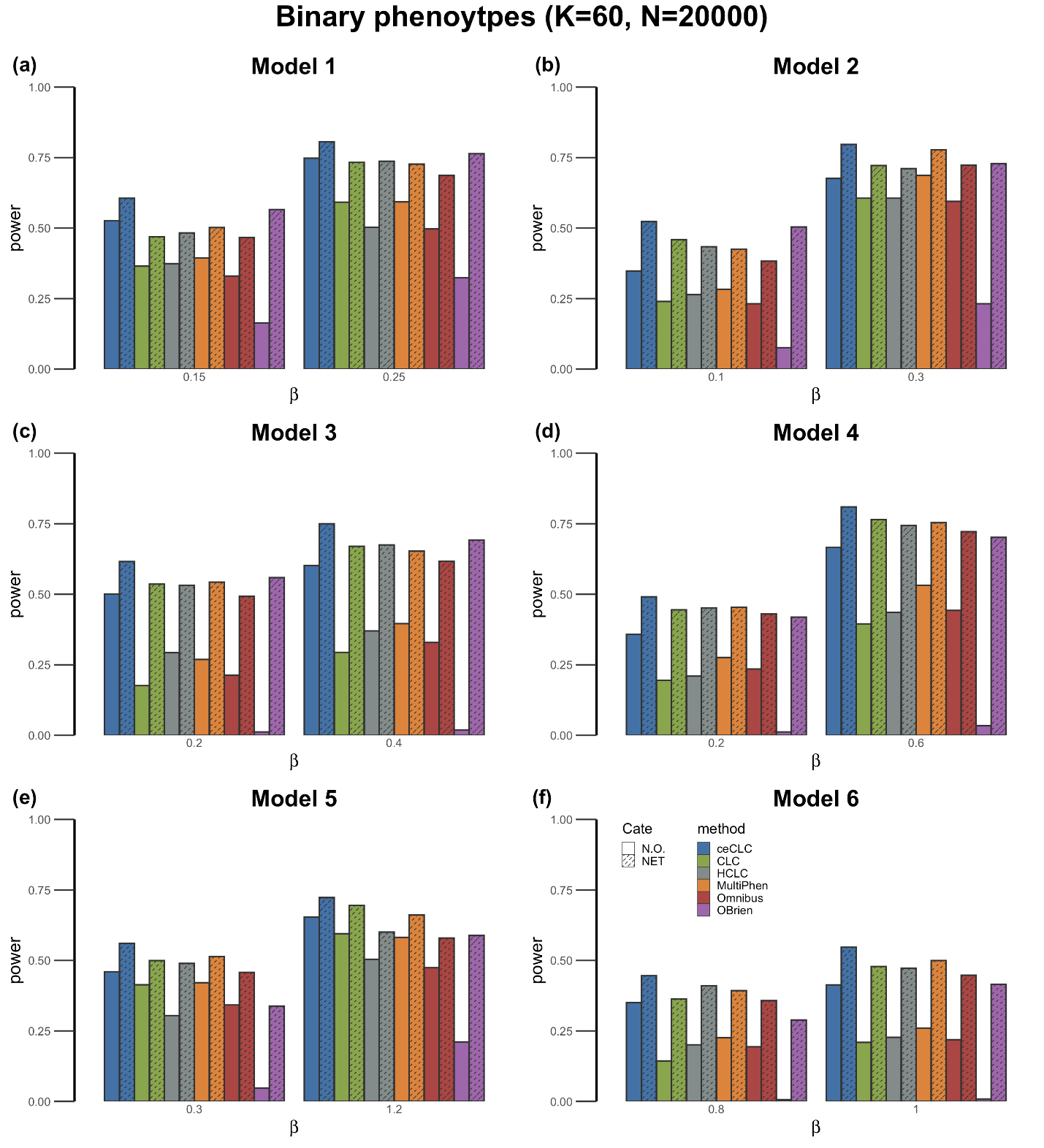
**

**Fig J**. Power comparisons of the six tests as a function of effect size $\beta$ under six models. The number of binary phenotypes (with extremely unbalanced case-control ratios) is 100 and the sample size is 10,000. The power of all of the six tests is evaluated using 10 MC runs.

**
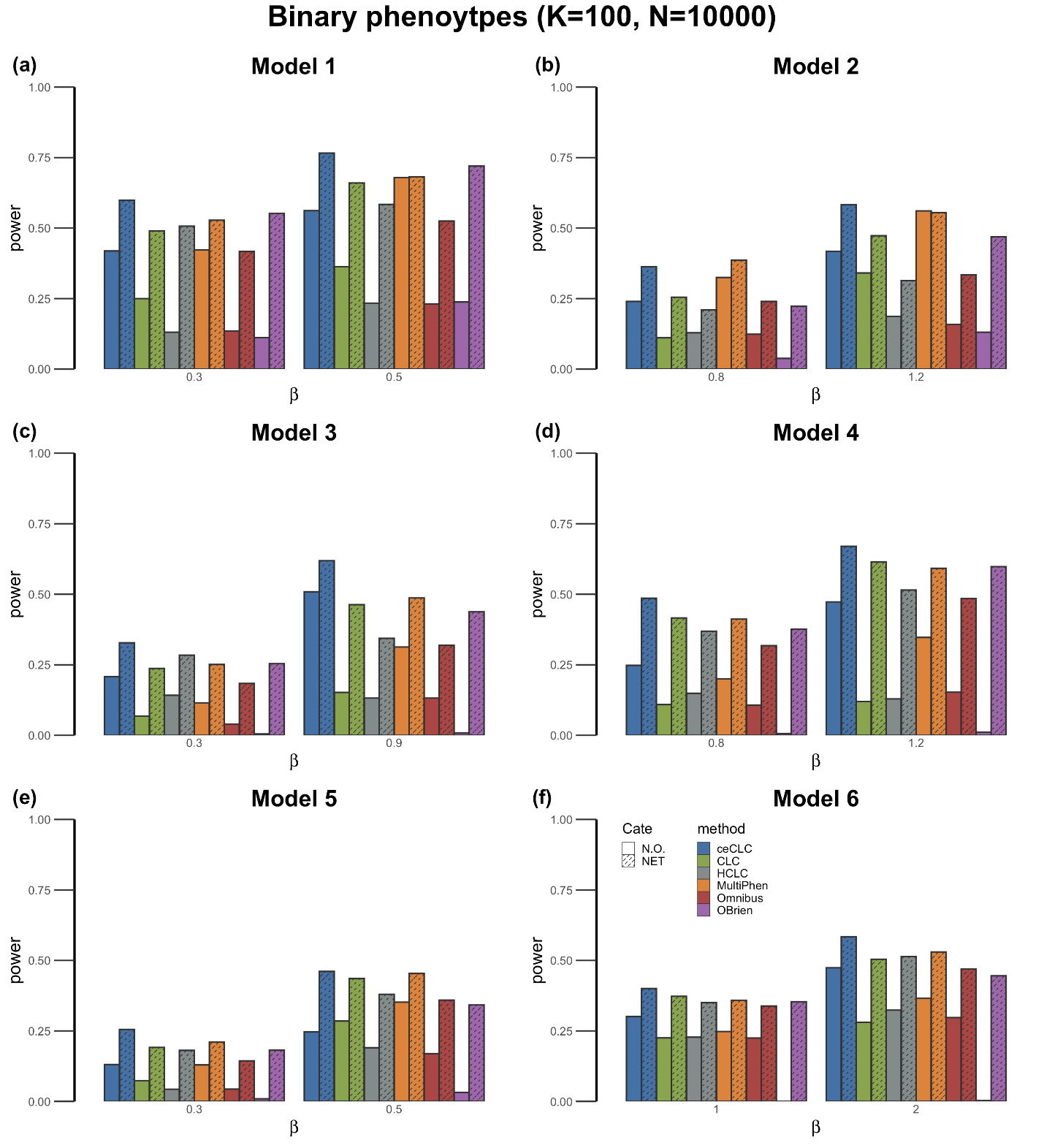
**

**Fig K**. Power comparisons of the six tests as a function of effect size $\beta$ under six models. The number of binary phenotypes (with extremely unbalanced case-control ratios) 100 and the sample size is 20,000. The power of all of the six tests is evaluated using 10 MC runs.

**
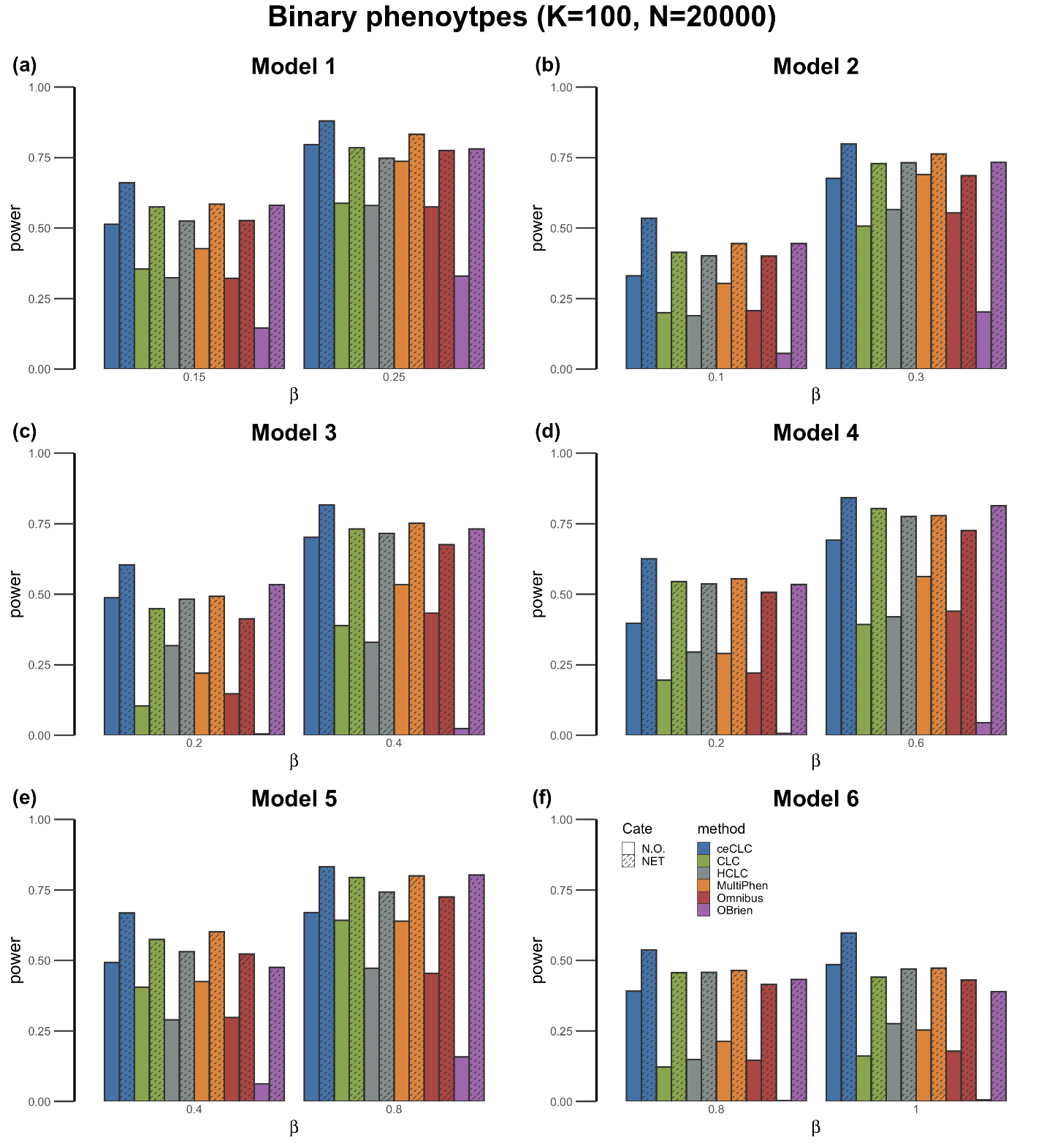
**

**Fig L**. Power comparisons of the six tests as a function of the sample size under the six models. The number of mixture phenotypes (half continuous phenotypes and half binary phenotypes with balanced case-control ratios) is 52 in the UK Biobank. The power of all of the six tests is evaluated using 10 MC runs.


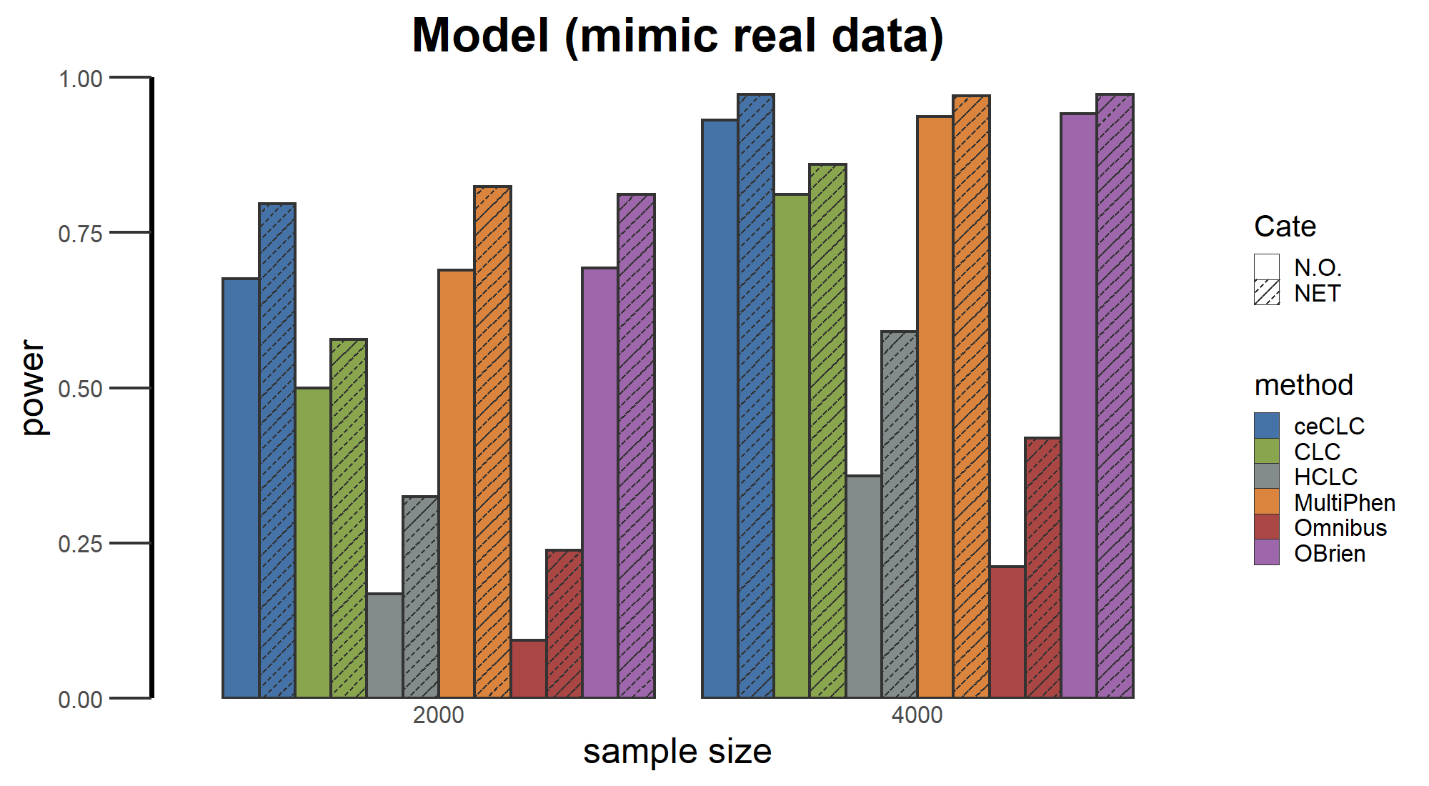


**Fig M**. The Manhattan plots of four different diseases based on the saddlepoint approximation. Systemic lupus erythematosus (M32.9), Sicca syndrome (M35.0), and Trigger finger (M65.3) are detected in Module III by our proposed GPN. Both Trigger finger (M65.3) and Synovitis and tenosynovitis (M65.9) are classified into the same ICD-codes category (M65). The horizontal red dashed line represents the threshold for commonly used genome-wide significance level $5\times10^{-8}$.


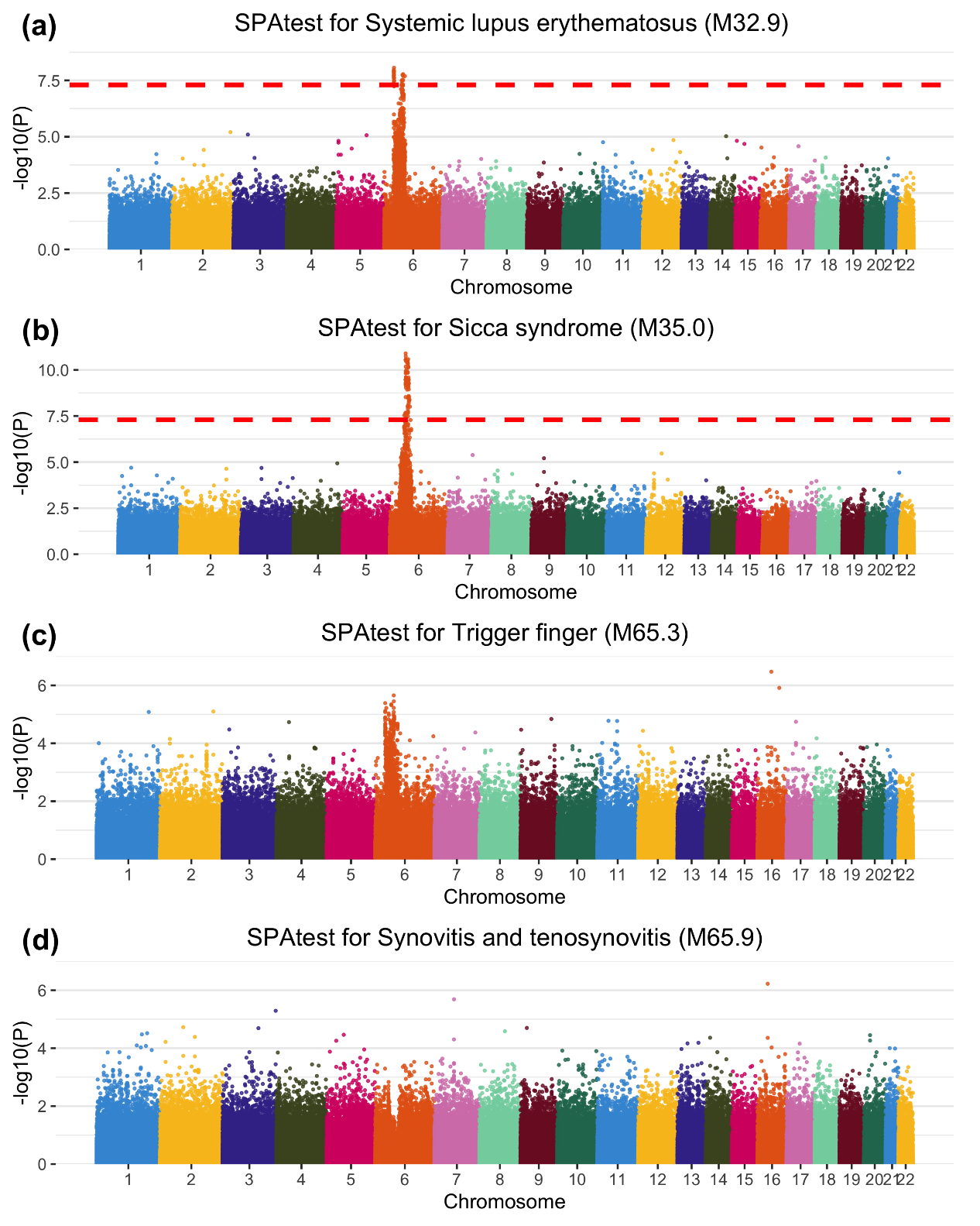


**Fig N.** Dendrogram of hierarchical clustering method based on the genetic correlation of phenotypes obtained by GPN and the phenotypic correlation estimated by LDSC, respectively.

**
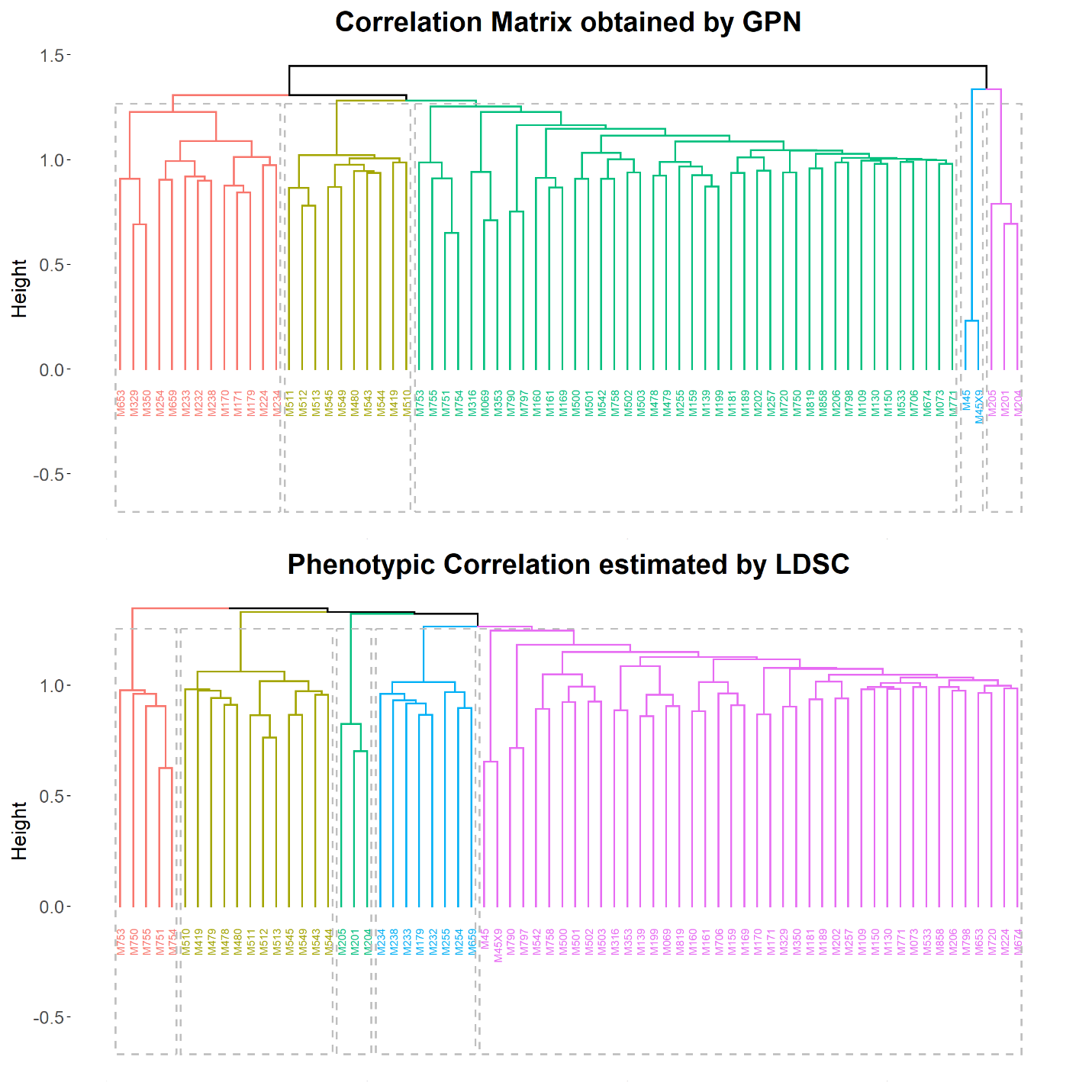
**

**Fig O.** Dendrogram of hierarchical clustering method based on the genetic correlation of phenotypes obtained by GPN and the genetic correlation estimated by LDSC, respectively.

**
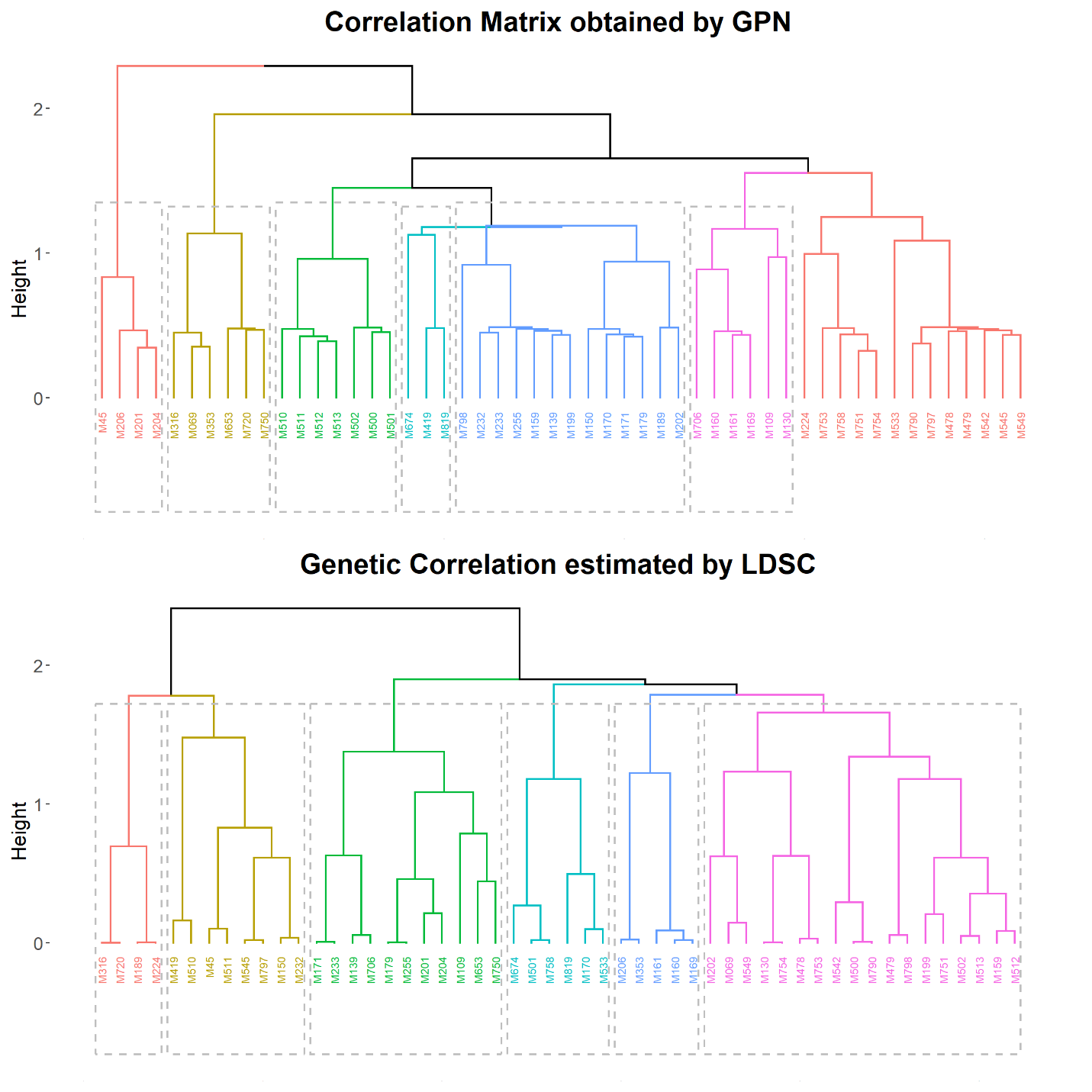
**

**Fig P.** The Venn diagrams of the number of significant SNPs identified by ceCLC, CLC, HCLC, O’Brien, and Omnibus in N.O. and NET.


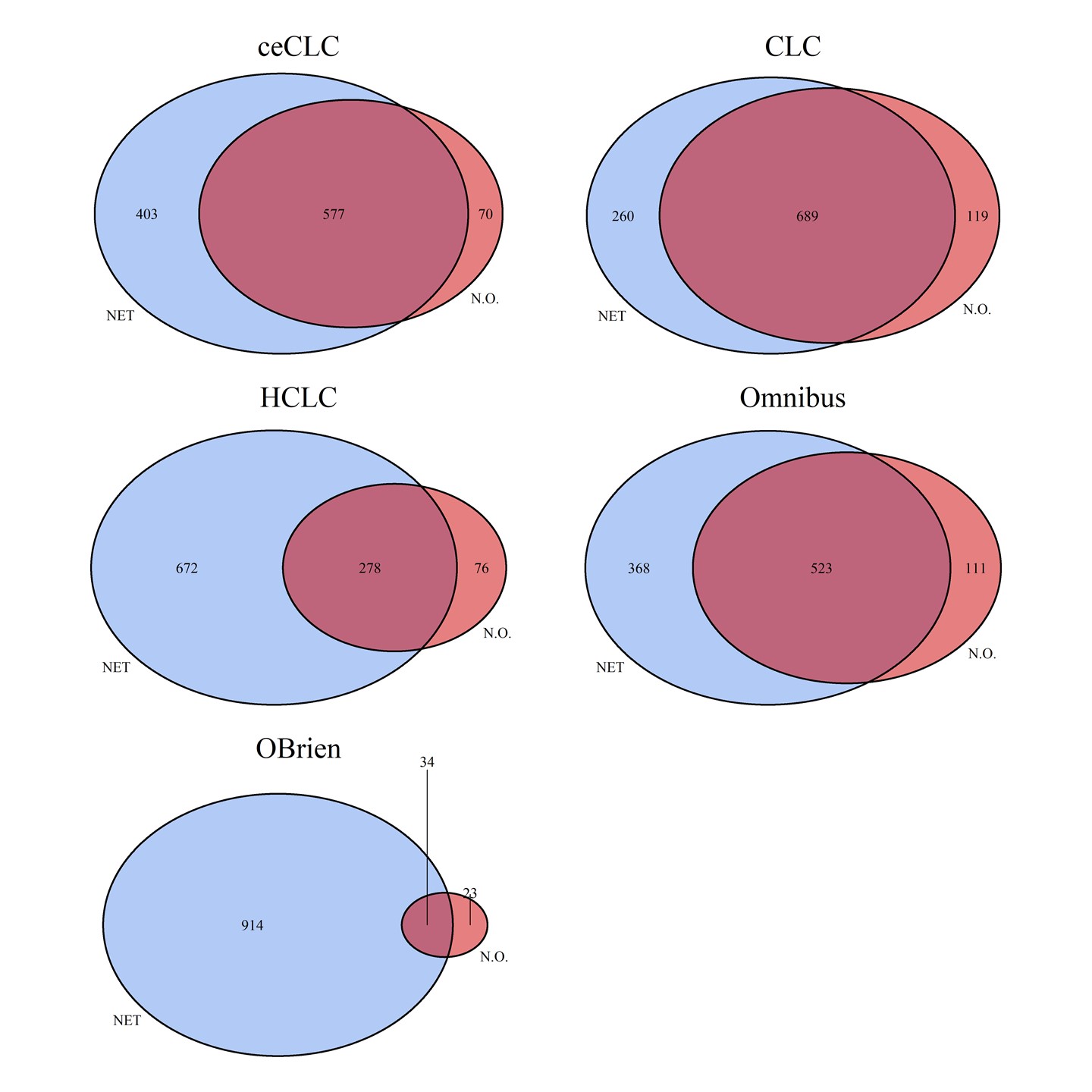


**Fig Q.** The QQ plots and inflation factors $\lambda$ in each of the eight network modules for different tests in the real data analysis.

**
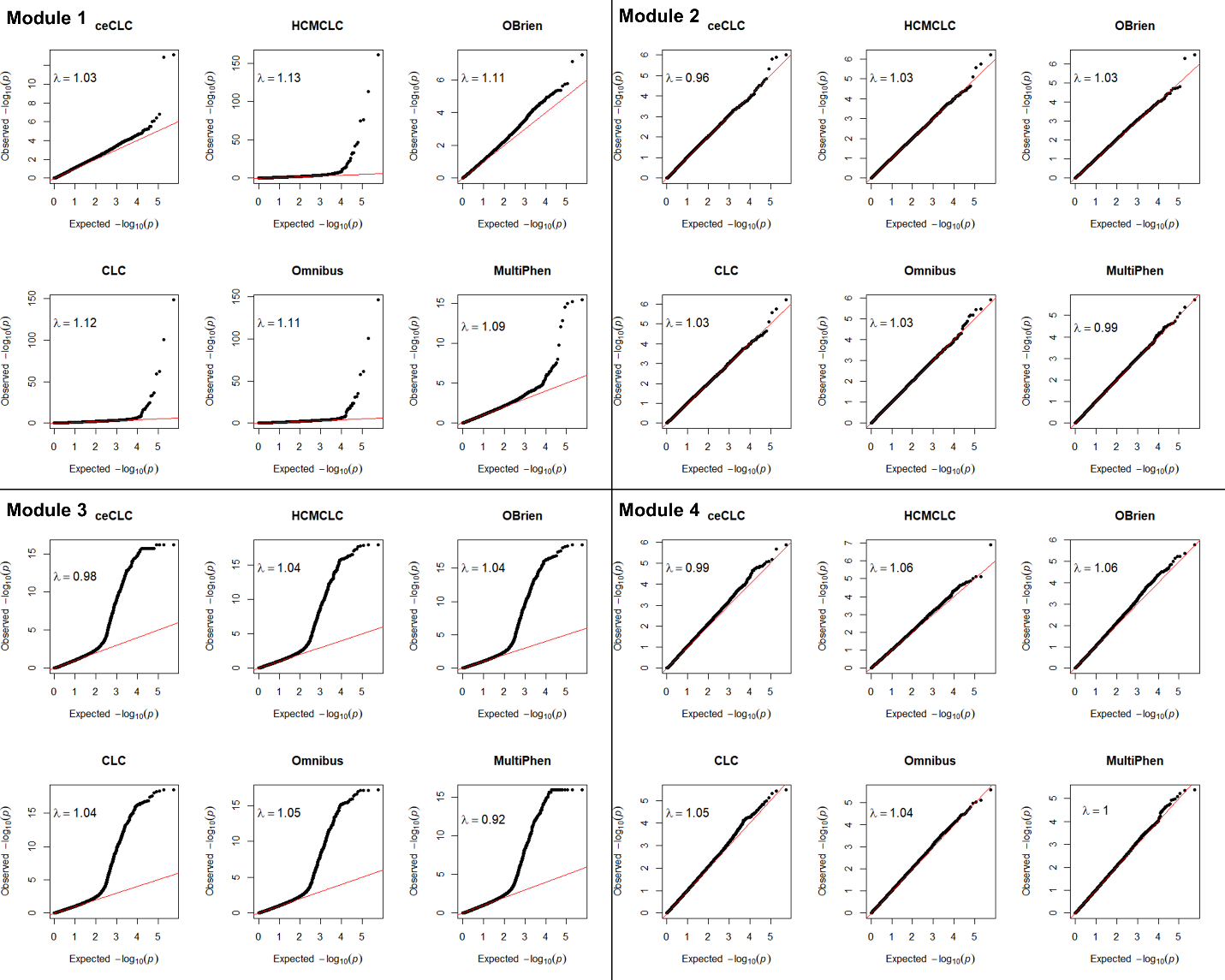
**

**
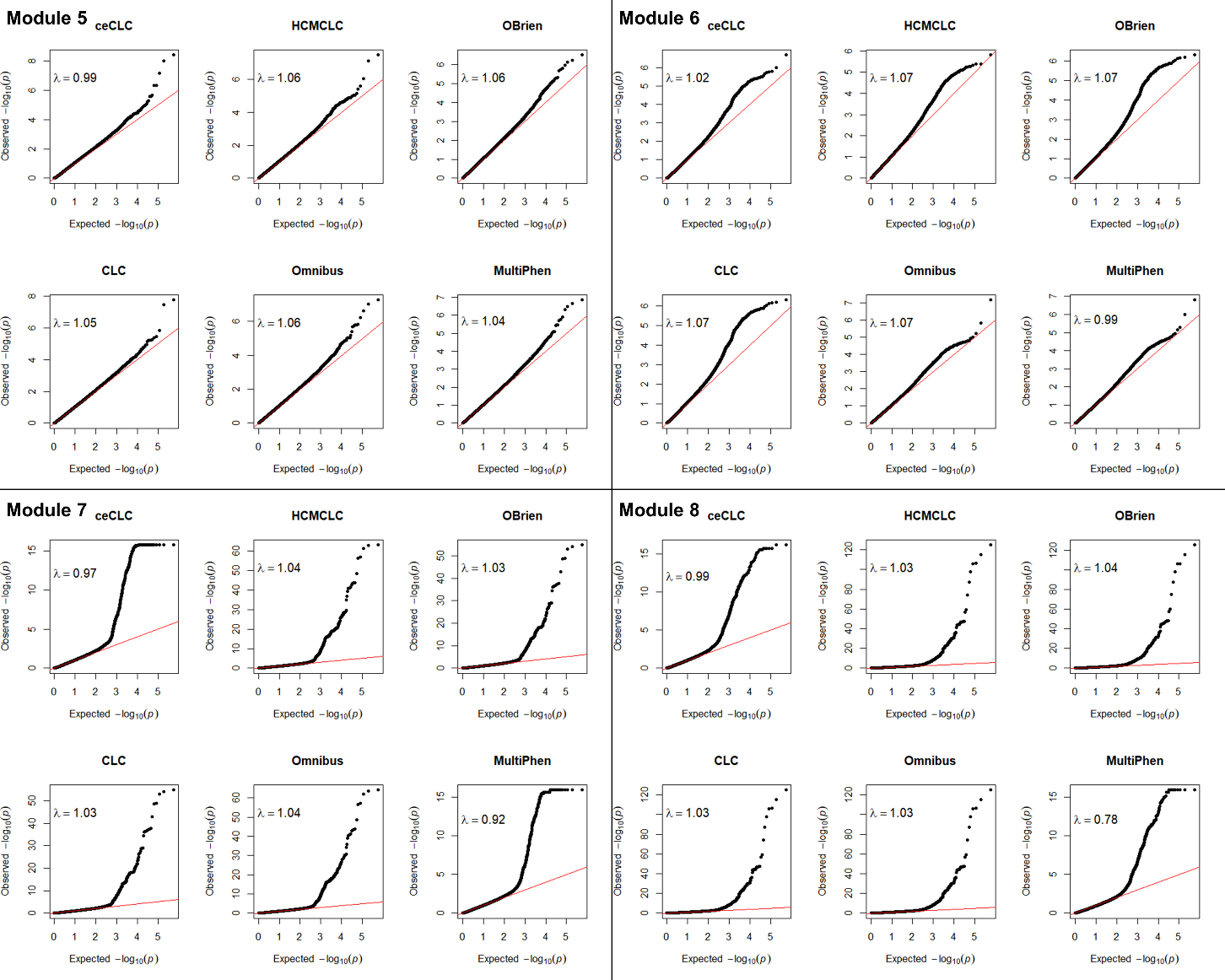
**

**Fig R.** Tissue expression analysis for mapped genes identified by ceCLC in N.O. (a) and NET (b), respectively.

**
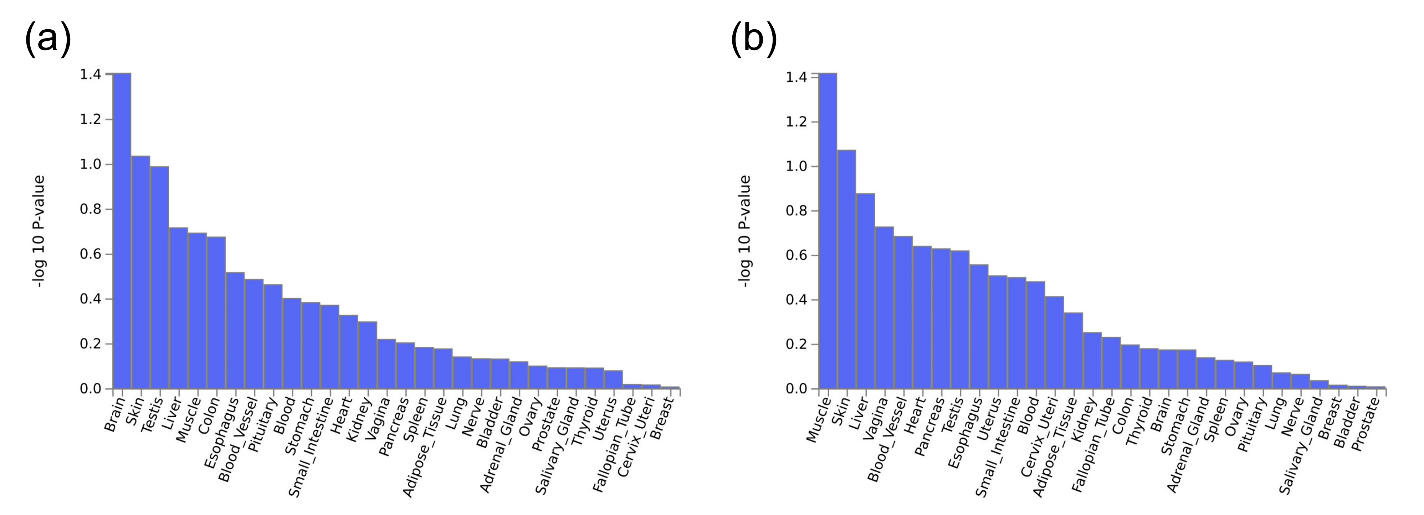
**

**Fig S**. Colocalization signals. Lead SNPs are selected for colocalization analysis when the top associated SNP identified by ceCLC was also associated with gene expression in the Muscle Skeletal tissue.

**
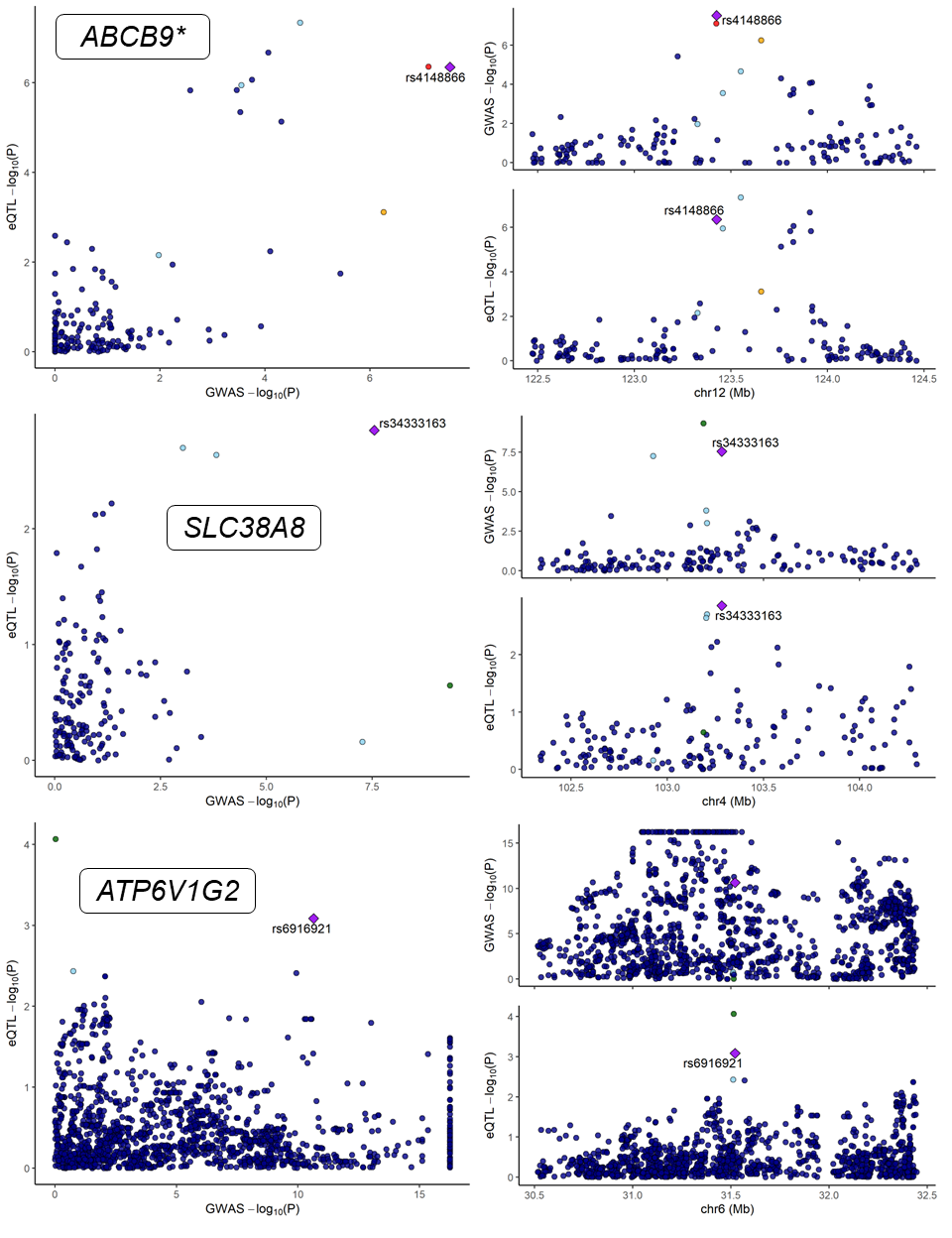
**

**Fig T**. Power comparisons of the six tests under six models. The number of mixture phenotypes (half continuous phenotypes and half binary phenotypes with balanced case-control ratios) is 60 and the sample size is 1,000. The power of all of the six tests is evaluated using 10 MC runs.


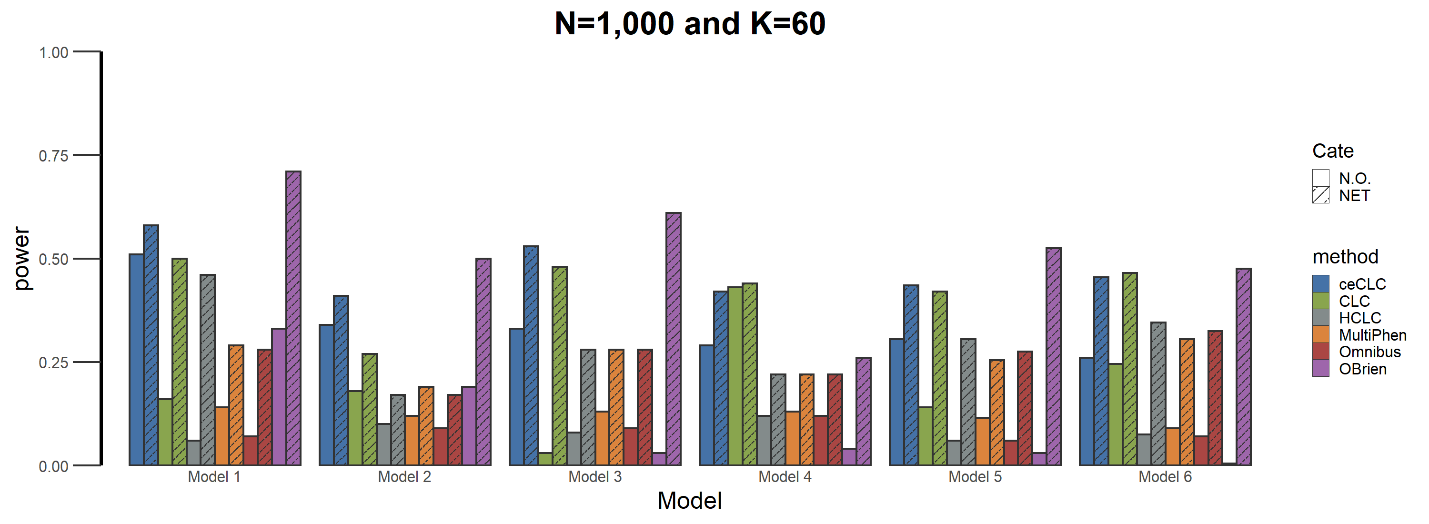


**Fig U**. Flow chart of UK Biobank data preprocessing. *Pre-process on phenotype:* i. Select White British subjects (White British); ii. Remove individuals who are marked as outliers for heterozygosity or missing rates (Low Heterozygosity); iii. Exclude individuals who have been identified to have ten or more third-degree relatives or closer (Not Three-degree Relatives); iv. Remove individuals having very similar ancestry based on a principal component analysis of the genotypes (Similar Ancestry); v. Remove individuals based on removal by the UK Biobank (Removal by the UK Biobank). *Quality controls (QCs) on genotype:* Filter out genetic variants, with i. Missing rate larger than 5% (“--mind 0.05”), ii. Hardy-Weinberg equilibrium exact test p-values less than $10^{-6}$ (“--hwe 1e-6”), iii. Minor allele frequency (MAF) less than 5% (“--maf 0.05”). We also filter out individuals, with iv. Missing rate larger than 5% (“--mind 0.05”) v. Individuals without sex (“--no-sex”).


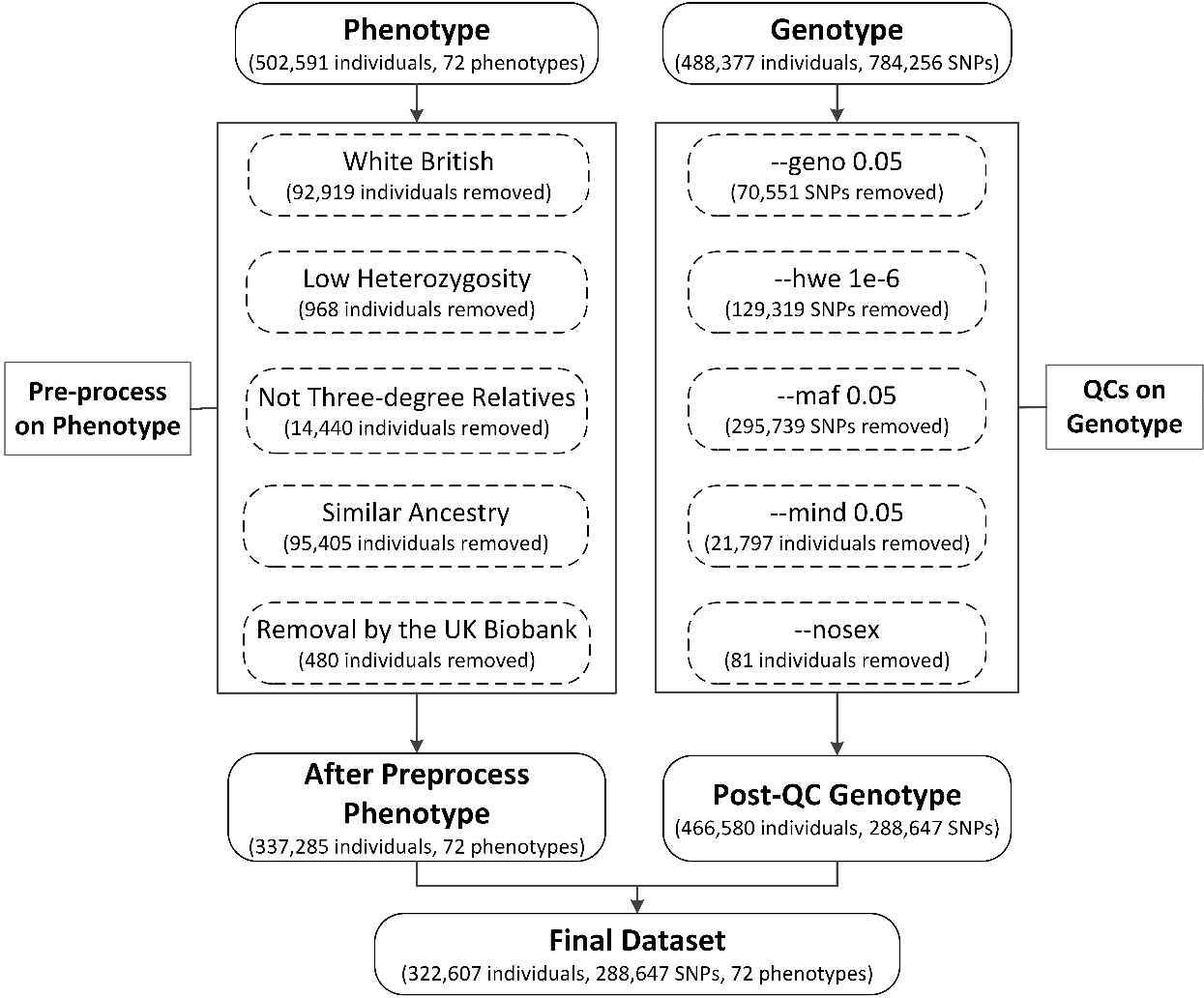


**References**

1. Sha Q, Wang Z, Zhang X, Zhang S. A clustering linear combination approach to jointly analyze multiple phenotypes for GWAS. Bioinformatics. 2019;35(8):1373-9.

2. Wang M, Zhang S, Sha Q. A computationally efficient clustering linear combination approach to jointly analyze multiple phenotypes for GWAS. PloS one. 2022;17(4):e0260911.

3. Liu Y, Xie J. Cauchy combination test: a powerful test with analytic p-value calculation under arbitrary dependency structures. Journal of the American Statistical Association. 2020;115(529):393-402.

4. Liu Y, Chen S, Li Z, Morrison AC, Boerwinkle E, Lin X. ACAT: a fast and powerful p value combination method for rare-variant analysis in sequencing studies. The American Journal of Human Genetics. 2019;104(3):410-21.

5. Liang X, Cao X, Sha Q, Zhang S. HCLC-FC: A novel statistical method for phenome-wide association studies. Plos one. 2022;17(11):e0276646.

6. Liang X, Sha Q, Rho Y, Zhang S. A hierarchical clustering method for dimension reduction in joint analysis of multiple phenotypes. Genetic epidemiology. 2018;42(4):344-53.

7. O’Reilly PF, Hoggart CJ, Pomyen Y, Calboli FC, Elliott P, Jarvelin M-R, et al. MultiPhen: joint model of multiple phenotypes can increase discovery in GWAS. PloS one. 2012;7(5):e34861.

8. O'Brien PC. Procedures for comparing samples with multiple endpoints. Biometrics. 1984:1079-87.

9. Dey R, Schmidt EM, Abecasis GR, Lee S. A fast and accurate algorithm to test for binary phenotypes and its application to PheWAS. The American Journal of Human Genetics. 2017;101(1):37-49.

10. Chang CC, Chow CC, Tellier LC, Vattikuti S, Purcell SM, Lee JJ. Second-generation PLINK: rising to the challenge of larger and richer datasets. Gigascience. 2015;4(1):s13742-015-0047-8.

11. Denny JC, Bastarache L, Roden DM. Phenome-wide association studies as a tool to advance precision medicine. Annual review of genomics and human genetics. 2016;17:353-73.

12. Sha Q, Wang X, Wang X, Zhang S. Detecting association of rare and common variants by testing an optimally weighted combination of variants. Genetic epidemiology. 2012;36(6):561-71.

13. Tachmazidou I, Hatzikotoulas K, Southam L, Esparza-Gordillo J, Haberland V, Zheng J, et al. Identification of new therapeutic targets for osteoarthritis through genome-wide analyses of UK Biobank data. Nature genetics. 2019;51(2):230-6.

14. Kim SK, Nguyen C, Jones KB, Tashjian RZ. A Genome Wide Association Study For Shoulder Impingement and Rotator Cuff Disease. Journal of Shoulder and Elbow Surgery. 2021.

15. Johnston KJ, Adams MJ, Nicholl BI, Ward J, Strawbridge RJ, Ferguson A, et al. Genome-wide association study of multisite chronic pain in UK Biobank. PLoS genetics. 2019;15(6):e1008164.

16. Aterido A, Cañete JD, Tornero J, Ferrándiz C, Pinto JA, Gratacós J, et al. Genetic variation at the glycosaminoglycan metabolism pathway contributes to the risk of psoriatic arthritis but not psoriasis. Annals of the Rheumatic diseases. 2019;78(3):355-64.

17. Bentham J, Morris DL, Graham DSC, Pinder CL, Tombleson P, Behrens TW, et al. Genetic association analyses implicate aberrant regulation of innate and adaptive immunity genes in the pathogenesis of systemic lupus erythematosus. Nature genetics. 2015;47(12):1457-64.

18. Kim SK. Identification of 613 new loci associated with heel bone mineral density and a polygenic risk score for bone mineral density, osteoporosis and fracture. PloS one. 2018;13(7):e0200785.

19. Hou S, Yang Z, Du L, Jiang Z, Shu Q, Chen Y, et al. Identification of a susceptibility locus in STAT4 for Behçet's disease in Han Chinese in a genome‐wide association study. Arthritis & Rheumatism. 2012;64(12):4104-13.

20. Renauer PA, Saruhan‐Direskeneli G, Coit P, Adler A, Aksu K, Keser G, et al. Identification of susceptibility loci in IL6, RPS9/LILRB3, and an intergenic locus on chromosome 21q22 in Takayasu arteritis in a genome‐wide association study. Arthritis & rheumatology. 2015;67(5):1361-8.

21. Allanore Y, Saad M, Dieudé P, Avouac J, Distler JH, Amouyel P, et al. Genome-wide scan identifies TNIP1, PSORS1C1, and RHOB as novel risk loci for systemic sclerosis. PLoS Genet. 2011;7(7):e1002091.

22. Chung SA, Brown EE, Williams AH, Ramos PS, Berthier CC, Bhangale T, et al. Lupus nephritis susceptibility loci in women with systemic lupus erythematosus. Journal of the American Society of Nephrology. 2014;25(12):2859-70.

23. Rothwell S, Cooper RG, Lundberg IE, Miller FW, Gregersen PK, Bowes J, et al. Dense genotyping of immune-related loci in idiopathic inflammatory myopathies confirms HLA alleles as the strongest genetic risk factor and suggests different genetic background for major clinical subgroups. Annals of the rheumatic diseases. 2016;75(8):1558-66.

24. Miller FW, Chen W, O'Hanlon TP, Cooper RG, Vencovsky J, Rider LG, et al. Genome-wide association study identifies HLA 8.1 ancestral haplotype alleles as major genetic risk factors for myositis phenotypes. Genes & Immunity. 2015;16(7):470-80.

25. Cordero AIH, Gonzales NM, Parker CC, Sokolof G, Vandenbergh DJ, Cheng R, et al. Genome-wide associations reveal human-mouse genetic convergence and modifiers of myogenesis, CPNE1 and STC2. The American Journal of Human Genetics. 2019;105(6):1222-36.

26. Johnson TA, Mashimo Y, Wu J-Y, Yoon D, Hata A, Kubo M, et al. Association of an IGHV3-66 gene variant with Kawasaki disease. Journal of human genetics. 2021;66(5):475-89.

27. Terao C, Yamada R, Ohmura K, Takahashi M, Kawaguchi T, Kochi Y, et al. The human AIRE gene at chromosome 21q22 is a genetic determinant for the predisposition to rheumatoid arthritis in Japanese population. Human molecular genetics. 2011;20(13):2680-5.
